# Tuning Overbias Plasmon Energy and Intensity in Molecular Plasmonic Tunneling Junctions by Atomic Polarizability

**DOI:** 10.1021/jacs.4c05544

**Published:** 2024-06-28

**Authors:** Wei Du, Xiaoping Chen, Tao Wang, Qianqi Lin, Christian A. Nijhuis

**Affiliations:** † Department of Chemistry, National University of Singapore, 3 Science Drive 3, 117543 Singapore, Singapore; § Fujian Provincial Key Laboratory of Modern Analytical Science and Separation Technology, College of Chemistry, Chemical Engineering and Environment, Minnan Normal University, Zhangzhou 363000, China; ∥ Hybrid Materials for Optoelectronics Group, Department of Molecules and Materials, MESA+ Institute for Nanotechnology, Molecules Center and Center for Brain-Inspired Nano Systems, Faculty of Science and Technology, University of Twente, 7500AE Enschede, The Netherlands

## Abstract

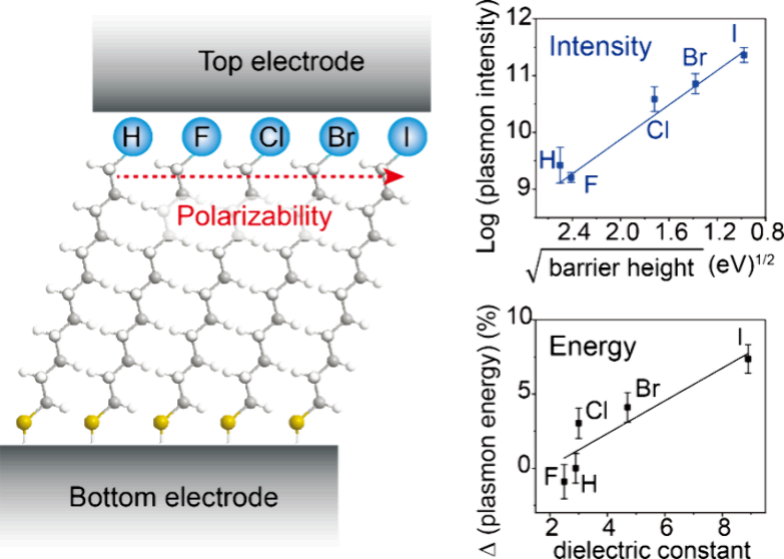

Plasmon excitation in molecular tunnel junctions is interesting because the plasmonic properties of the device can be, in principle, controlled by varying the chemical structure of the molecules. The plasmon energy of the excited plasmons usually follows the quantum cutoff law, but frequently overbias plasmon energy has been observed, which can be explained by quantum shot noise, multielectron processes, or hot carrier models. So far, clear correlations between molecular structure and the plasmon energy have not been reported. Here, we introduce halogenated molecules (HS(CH_2_)_12_X, with X = H, F, Cl, Br, or I) with polarizable terminal atoms as the tunnel barriers and demonstrate molecular control over both the excited plasmon intensity and energy for a given applied voltage. As the polarizability of the terminal atom increases, the tunnel barrier height decreases, resulting in an increase in the tunneling current and the plasmon intensity without changing the tunneling barrier width. We also show that the plasmon energy is controlled by the electrostatic potential drop at the molecule–electrode interface, which depends on the polarizability of the terminal atom and the metal electrode material (Ag, Au, or Pt). Our results give new insights in the relation between molecular structure, electronic structure of the molecular junction, and the plasmonic properties which are important for the development of molecular scale plasmonic-electronic devices.

## Introduction

Plasmonics has a wide range of applications in catalysis, sensing, subwavelength imaging, plasmon enhanced spectroscopy and lasing.^1−5^ Plasmons at interfaces can be excited not only by photons but also by high energy electrons in transmission electron microscopy (TEM)^6−8^ or low energy tunneling electrons in scanning tunneling microscopy^9−11^ and metal–insulator–metal (MIM) tunnel junctions^12−24^ via inelastic tunneling (IET) which may find applications as nanoscale light sources, data processing devices, or integrated optical circuits. Plasmonic molecular junctions are interesting for in operando single-molecule Raman spectroscopy,^25,26^ controlled plasmonic grafting of molecular junctions,^27^ and hot carrier generation.^28−31^ We have shown that the generated plasmons can be controlled at the molecular level,^16^ where orientation of the molecules modulates polarization,^32^ and the structure of the molecules determines intensity and the dynamics^33^ of the light emission. However, molecular plasmonic tunneling junctions are still not clearly understood for several reasons including: (i) the origin of over bias emission is still unclear,^28,34−42^ (ii) the shape of the tunneling barrier is usually assumed to be rectangular (following the Simmons model) which is probably not justified (especially when dipoles or polarizable moieties^43−45^ are present), and (iii) large voltages^46,47^ (≫3 V) are applied which can lead to photon emission unrelated to excitation by IET (such as heating^39,40^). Here we show, by simply changing one terminal atom in the molecular chemical structure, that the polarizability α of the terminal atom modifies the tunnel barrier shape and electrostatic potential profile in the molecular junction, which then affects both plasmon intensity (by 2 orders of magnitude) and overbias plasmon emission energy (over a range of 0.2 eV) emitted from these junctions without changing the barrier width at low *V* of ≤2.4 V.

Light emission from tunneling junctions has been known since the 1970s^12^ and, recently, the plasmonic properties of tunneling junctions have been controlled via optical antennas.^13−15,18,19,23,24^ From an electronics point of view, both the tunnel barrier width *d* and tunnel barrier height φ affect the electrical properties of MIM junctions, as they determine the tunneling rate across the junction and hence the plasmon intensity.^48−50^ Although how the shape of the junction affects the plasmonic properties has been frequently investigated,^48^ it is unclear to which extent the barrier properties affect the plasmonic properties. Often the shape of the tunneling barrier is modeled as a simple rectangular barrier using the Simmons model (see Background in the Supporting Information), but this kind of analysis is too simplistic to ignore; for instance, the presence of interface dipoles,^51,52^ image charges,^53−55^ and Fermi level pinning^56,57^ all affect the shape of the tunneling barrier. In molecular junctions, the shape of the tunneling barrier can be quite complex depending on the presence of redox-active groups,^58^ molecular dipoles,^51,52^ or polarizable groups,^43−45^ all of which can screen the electric field and thus alter the energy that is available for the tunneling charge carriers to excite plasmons.

All of these considerations complicate our understanding of the relation between *d* and φ and the energy of the plasmons. More specifically, for plasmon excitation by tunneling electrons, the energy of the photons (*hv*) that escapes the junction should not be higher than the energy provided by the bias *V* across the junction following the quantum cutoff law^12^ given by eq 1
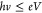
1where *h* is Planck’s constant, *v* is the frequency of the photon, and *e* is the elementary charge. Indeed, most studies report cutoff photon energies *E*_c_ ≈ *eV*,^9−24^ but exceptions with *E*_c_ > *eV*(28,34−42) or *E*_c_ < *eV*(46,47) have also been observed. The underlying mechanisms that cause these deviations are not fully disclosed. For instance, photon emission above quantum threshold (*E*_c_ > *eV*; see Background in the Supporting Information) has been explained using quantum shot noise,^34−36^ multielectron processes,^37,38^ and hot carrier based emission.^28,39−42^ McCreery and co-workers attributed the subthreshold light emission (*E*_c_ < *eV*) to inelastic energy loss of the charge carriers during charge transport along the molecular chain although the mechanism of the light emission in their experiments is unclear considering the high applied bias up to 9 V.^46,47^

Here we report molecular electronic plasmon sources based on self-assembled monolayer (SAM) tunnel junctions (STJs) where the shape of the tunneling barrier and electrostatic potential profile (see the Background in the Supporting Information) are determined by the properties of the SAM and the metal electrodes. We systematically changed α of the terminal group of the SAM, thereby changing the tunnel barrier shape and the effective potential drop along the SAM. We show that the plasmon energy (or *E*_c_) can be related to the electrostatic potential profile of the molecular tunneling barrier φ, which includes effective potential drop along the SAM, and the built-in fields *V*_int_ that are always present at metal–insulator interfaces due to the interface dipoles.^51,52^ To study the role of changes in work functions and Fermi-level pinning, we changed the material of the bottom electrode and found that *E*_c_ changes with the work function shift ΔΦ (the shift of metal work function after modification of SAM, Pt > Au > Ag). Our results show that *E*_c_ directly relates to the potential drop and electrostatic potential profile of the junctions, leading to overbias emission of up to 0.2 eV, which also gives new insights into the plasmon excitation mechanism.

## Results and Discussion

### Experimental Design of the Plasmonic Junctions

**Figure 1:**
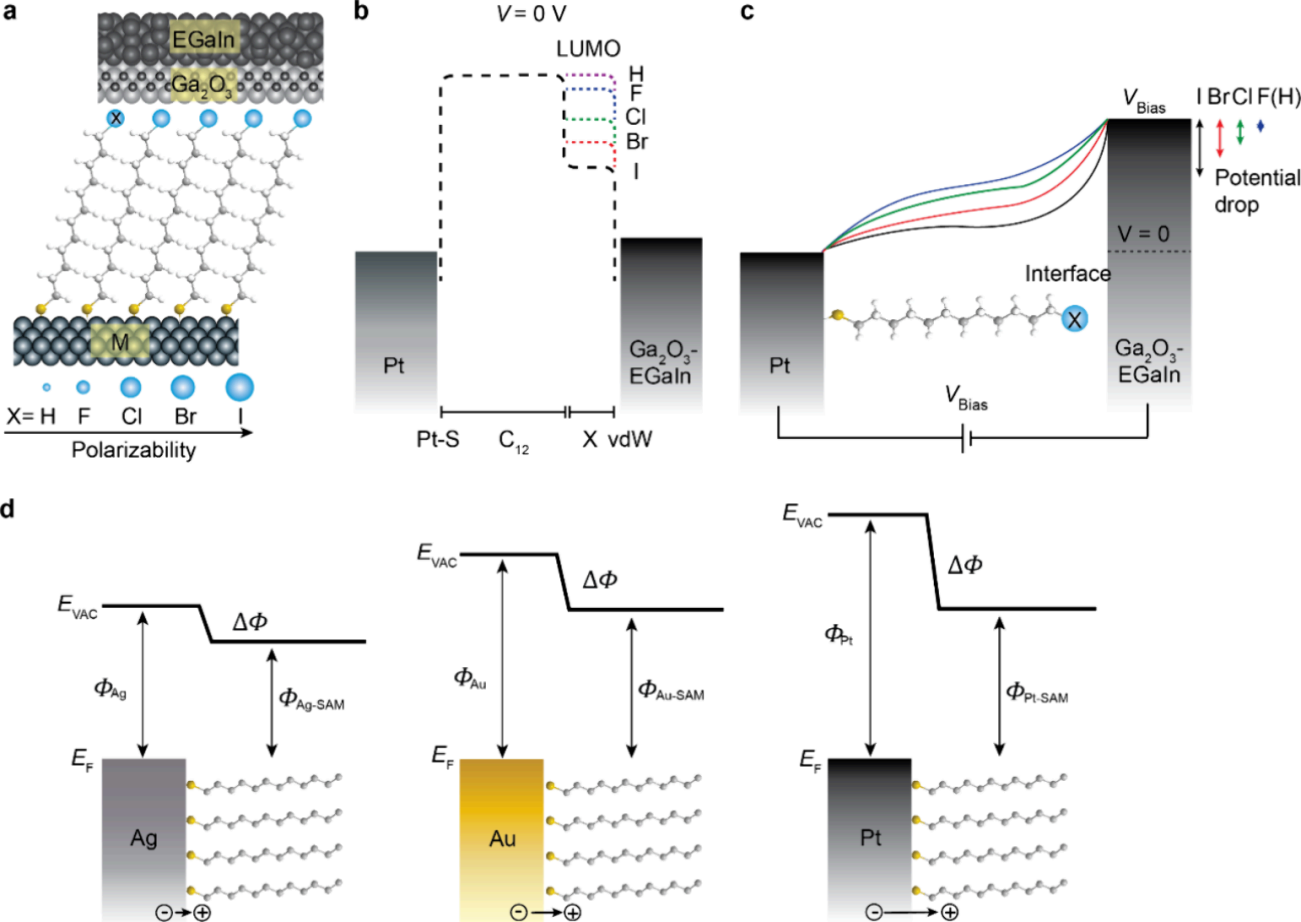
(a) Schematic representation of the STJs with SC_12_X (X = H, F, Cl, Br, or I) SAMs on different metal substrates (M = Ag, Au, or Pt). (b) Energy level diagram of the Pt-SC_12_X//EGaIn junctions with zero bias, where X is hydrogen or halogen, as stated in panel a. The shapes of the tunnel barriers are indicated by the dashed lines which change as a function of X. (c) The shape of the electrostatic potential profiles across the Pt-SC_12_X//EGaIn junctions (EGaIn stands for eutectic alloy of gallium and indium) with *V*_Bias_ to the EGaIn top electrode (in all of our experiments, the bottom electrode was grounded). (d) Interface energetics of the SC_12_-bottom electrode interface. Φ_M_ and Φ_M-SAM_ are work functions of the bottom electrodes before and after SAM formation. The black arrows indicate the relative magnitude of the molecular interface dipoles.

Figure 1a shows a schematic of the STJs. We used template-stripped bottom-electrodes of Ag, Au, or Pt to support the SAMs of S(CH_2_)_12_X (in short SC_12_X) where X = H, F, Cl, Br, or I and formed electrical top contacts to these SAMs of EGaIn with the well-established EGaIn technique^59^ (EGaIn = eutectic gallium indium alloy). Although Pt is a more lossy plasmonic material than Au or Ag,^60,61^ plasmons on Pt have been excited by both optical^62^ and electrical^63^ means. Besides, junctions based on Pt bottom electrodes have a higher breakdown voltage than the Au and Ag counterparts^64^ which facilitate the optical measurements in the high bias regime. Therefore, we focus here on Pt substrate where we systematically change the type of the atom of the headgroup X (to ensure to keep supramolecular changes to the monolayer structure negligible). Figure 1b shows the corresponding energy level diagram of these SC_12_X junctions on Pt where the shape of the tunnel barrier is indicated by the dashed lines. With this series of SAMs, *d* is kept similar, while φ decreases from F to I by ∼5 eV^43^ as α of SC_12_X increases as shown in Figure 1b (see below for details). Polarizable terminal atoms screen electric fields more efficiently than nonpolarizable ones as α directly relates to the dielectric constant (as, for instance, given by the Clausius-Mossotti relation^65^), which, in turn, affects the ionization potential (or electron affinity). We have shown elsewhere that indeed the relative dielectric constant ε_*r*_ of these junctions increases from 2.5 to 8.9 as X goes from F to I, enhancing the tunneling rates by 4 orders of magnitude, thus effectively reducing the effective tunneling barrier height.^43^ We hypothesize that, as the electric-field screening capability of the halogens increases, the potential drop at the SAM//EGaIn interface increases (Figure 1c) which effectively reduces the voltage, *V*_eff_, leading to a reduced excited plasmon energy (see below).

On the other hand, with the same SAM, we also changed the material of the bottom electrode and thus changed the interface energetics (see Background in the Supporting Information) at the SAM-bottom electrode interface. As shown in Figure 1d, although the native metals have different work functions Φ_M_, after SAM formation, the modified work functions Φ_M-SAM_ are similar due to Fermi-level pinning.^56,57^ This Fermi-level pinning results in a work function shift ΔΦ (Pt > Au > Ag) and associated formation of interface dipoles. These molecular interface dipoles are indicated by the black arrows. We hypothesize that *V*_int_ will also affect *E*_c_ (see below).

### Characterization of the SAMs

The SAM precursors HSC_12_X and the corresponding SAMs on Au, Ag, and Pt were prepared using previously reported methods (see Method section in the Supporting Information for details).^43^ The SAMs derived from HSC_12_X with X = H on Ag, Au, and Pt are well-known and their characterization has been reported before.^59,66−68^ The SAMs derived from HSC_14_X on Ag have also been reported.^43^ Below we have confirmed by X-ray photoelectron spectroscopy (XPS) that all SAMs have a similar surface coverage (Γ_SAM_, in nmol·cm^–2^) on Pt (Table 1 and the Method in the Supporting Information). In agreement with previous reports,^43,45^ we conclude that our SAMs formed well-organized and densely packed structures.

**Table 1:** Properties of the SC_12_X SAMs on Pt Substrates

SAMs	Γ_SAM_^a^ [nmol·cm^–2^]	Φ_Pt-SAM_ [eV]	HOMO onset [eV]	HOMO^b^ [eV]	*E*_g_^c^ [eV]	LUMO^c^ [eV]	φ^d^ [eV]	α^e^ [Å^3^]
SC_12_H	0.95	4.67	3.69	–8.36	9.92	1.56	6.23	4.50
SC_12_F	1.1	4.89	3.26	–8.15	9.05	0.90	5.79	3.74
SC_12_Cl	0.98	5.01	3.54	–8.55	6.51	–2.04	2.97	14.6
SC_12_Br	1.1	5.03	3.44	–8.47	5.35	–3.12	1.91	21.0
SC_12_I	0.81	5.02	3.34	–8.36	4.31	–4.05	0.97	32.9

a Γ_SAM_ is the relative surface coverage obtained from relative intensities of the Pt 4f peak vs that of SC_12_Br (with a surface coverage of 1.1 nmol·cm^–2^ according to ref (43)).

b HOMO was obtained by adding UPS measured Φ_Pt-SAM_ and HOMO onset. Systematic error is ±0.05 eV.

c LUMO was obtained by adding *E*_g_ (taken from ref (43)) to the HOMO energy. Systematic error is ±0.05 eV.

d φ was calculated as the energy offset between Φ_Pt-SAM_ and LUMO.

e α is the atomic polarizability for the terminal halogen atoms taken from ref (69).

With a similar HOMO energy, the LUMO energy decreases from X = F to X = I, which verifies the tunnel barrier shape, as shown in Figure 1b (and Supporting Information Figure S10). In other words, the tunneling barrier height (which is defined as the offset in energy between the LUMO and Fermi-level of the electrode) decreases from X = F to X = I. On the other hand, previous impedance measurements have shown increased ε_*r*_ by 3.5 times from X = F to X = I,^43^ as α is directly related to ε_*r*_ according to the Clausius-Mossotti relation.^65^ Thus, polarizable terminal atoms (e.g., X = I) screen electric fields more efficiently than nonpolarizable atoms (e.g., X = F), which changes the electrostatic potential profile as sketched in Figure 1c with larger potential drop for X = I than that for X = F at the monolayers–top electrode interface.

### Shift in Work Function and Interface Dipoles

**Figure 2:**
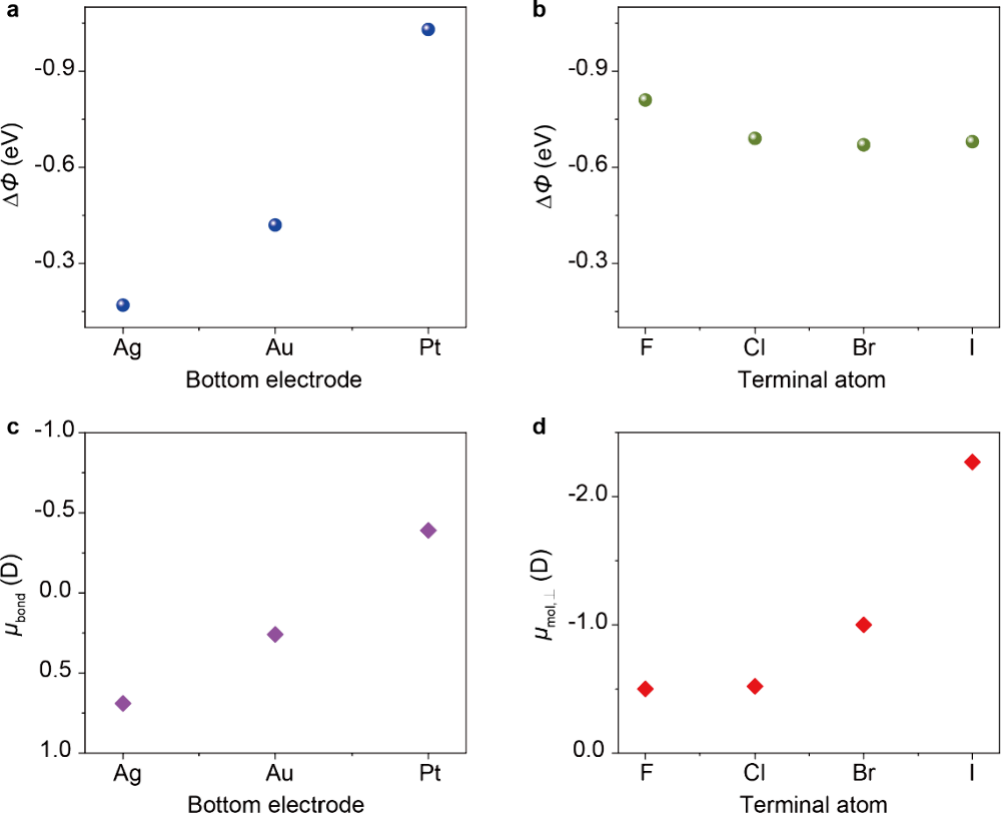
(a) Plot of ΔΦ for SC_12_H SAM on Ag, Au, and Pt. (b) Plot of ΔΦ for SC_12_X (X = F, Cl, Br, and I) SAMs on Pt. (c) Plot of μ_*bond*_ for SC_12_H SAM on Ag, Au, and Pt. (d) Plot of μ_*mol*,⊥_ for SC_12_X (X = F, Cl, Br, and I) SAMs on Pt.

When molecules are brought into contact with the metal surface, metal–molecule bonds form (see the Background in the Supporting Information), leading to changes in the energy-level alignment and formation of interface dipoles. With ultraviolet photoelectron spectroscopy (UPS; see Method section in the Supporting Information), we determined the Φ_M-SAM_ (eV) using well-established methods.^43^ The work function change ΔΦ of the metal surfaces induced by the modification of SAMs can be calculated from the difference between Φ_M-SAM_ and Φ_M_, which directly reflects the electrostatic potential steps at the SAM-bottom electrode interface (as drawn in Figure 1d). The interface dipole is determined by both μ_bond_ (the dipole of metal–sulfur M–S bond) and μ_mol,⊥_ (the dipole of molecule along the surface normal) given by the Helmholtz equation (eq 2)
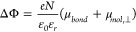
2where *e* is the elementary charge (1.602 × 10^–19^ C), *N* is the dipole density (here it corresponds to Γ_SAM_, in nmol·cm^–2^), μ_*bond*_ and μ_*mol*,⊥_ are the bond and molecular dipole moments (along surface normal direction, in D), ε_0_ is the vacuum permittivity (8.85 × 10^–12^ F/m), and ε_*r*_ is the relative dielectric constant. Since we varied the SAM and the bottom electrode separately, we can extract both the values of μ_*bond*_ and μ*_mol_*_,⊥_ for each terminal group X (see Method section in the Supporting Information, Table S1 and Table S2). Figure 2 shows the plot of ΔΦ, μ_*bond*_, and μ_*mol*,⊥_ for SC_12_H SAM on Ag, Au, and Pt substrates and for SC_12_X (X = F, Cl, Br, and I) SAMs on Pt substrates (see Method in Supporting Information for more details of the UPS analysis). According to Figure 2a, a clear trend from different metals in the value of ΔΦ (varying with Φ_M_) is observed. The value of ΔΦ increases from −0.17 to −1.03 eV when M changes from Ag to Pt. This change in ΔΦ offsets the overall change in the work function. In other words, the work function of all three SAM coated metals is essentially the same which is well-known and is caused by the push-back effect (or Fermi-level pinning).^56^ Further, due to the different nature of the M–S bond, the Au–S bond shows the smallest value of μ*_bond_* while Ag–S and Pt–S bonds show larger values of μ_bond_ but with different signs (Figure 2c), indicating the opposite direction of the two bond dipoles.^70^ For SAMs with different terminal atoms, the extracted μ_*mol*,⊥_ values change from −0.5 to −2.27 D within experimental error (Figure 2d).

### Electrical Characterizations of the STJs

**Figure 3:**
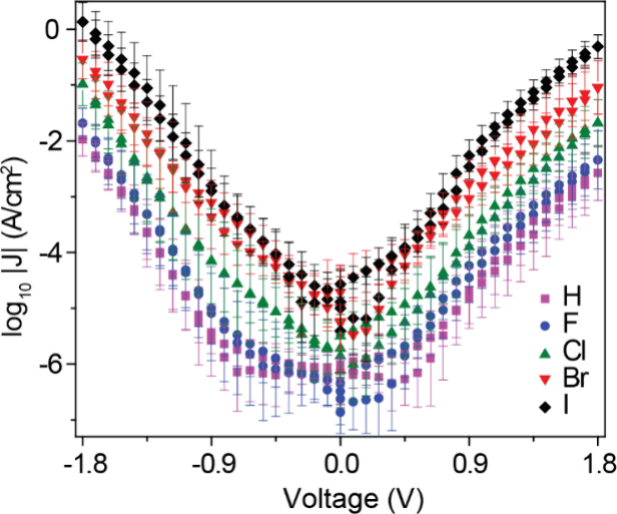
*J*(V) characterization of the STJs with SC_12_X SAMs at ±1.8 V on a Pt substrate. The error bars stand for the log-standard deviations.

To form the STJs, we used the EGaIn technique with the EGaIn electrode stabilized in a through-hole in a microfluidic network made from PDMS (polydimethylsiloxane) following a previously reported procedure^59^ (see Methods in the Supporting Information). We characterized these halogenated junctions electrically by collecting 50–200 *J*(V) curves for each type of SAM. Figure 3 shows the log-average *J*(V) curves of the junctions with SAMs of SC_12_X on Pt substrates recorded in the bias range of ±1.8 V (see Methods in the Supporting Information for details). The value of *J* increases by 2 orders of magnitude when changing from X = F or H to X = I in agreement with previously reported results.^43,45^ This increase in *J* is expected since the value of φ decreases for junctions with SAMs from X = F to I.

### Excited Plasmon Intensity

**Figure 4:**
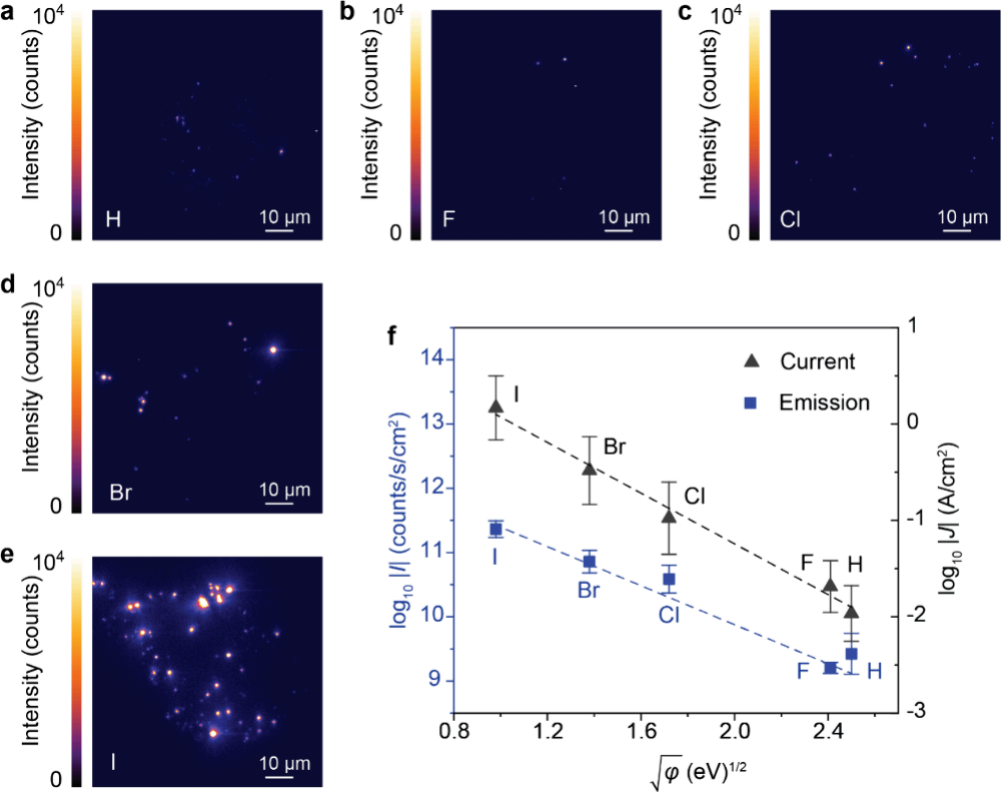
Representative real plane emission images at −1.8 V with SC_12_H (a), SC_12_F (b), SC_12_Cl (c), SC_12_Br (d), and SC_12_I (e) SAMs. (f) log_10_ |*J*| and log_10_ |*I*| at −1.8 V plot as a function of . The dashed lines are linear fits to the data, confirming the dependency of the light emission intensity and tunneling current on the barrier height.

To study the dependency of excited plasmon on the shape of the molecular tunnel barrier, we characterized the light emission, i.e., radiative decay of surface plasmons excited in the STJs, in the far field using an inverted optical microscope equipped with an electron multiplying charge-coupled device (EMCCD) and a 100× oil immersion objective with numerical aperture NA = 1.49 following previously reported procedures.^16^ Knowing that the tunneling current increases for junctions with SAMs from X = F to I (Figure 3), we measured the light emission images from the area of the STJ with an integration time of 2 min for junctions with low currents with X = F and H or 30 s (to avoid the saturation of the detector) for the other three conductive junctions with X = Cl, Br, or I. Figure 4a–e shows the representative real plane emission images recorded at −1.8 V. The light emission originates from discrete spots due to the high surface roughness of the top electrode, resulting in a much lower effective electrical contact area than the geometrical footprint of the top electrode. Since tunneling currents decay exponentially with distance, light emission is only observed in the effective electrical contact regions (see ref (66) for details). These images also show that the photon emission intensity increases when X changes from F or H to I. This follows the same trend of the above-mentioned increasing *J* and decreasing φ, induced by increasing α of the same series of X. In these STJs, the HOMO orbital is pinned to the bottom electrode; thus, φ (Table 1) can be calculated as the difference between the measured Φ_M-SAM_ (eV) and the calculated LUMO energy, and  should have a linear relationship with log_10_ |*J*| and hence log_10_ |*I*| based on eq S1 (see Background in Supporting Information), as shown in Figure 4f.

Here, the photon emission density *I* (in counts/s/cm^2^) is defined as the total photon counts divided by the integration time and geometric contact area of EGaIn. The value of *J* and photon emission density *I* (in counts/s/cm^2^) for each SAM was determined at −1.8 V (see Methods in the Supporting Information). Figure 4f shows that both log_10_ |*J*| and log_10_ |*I*| decrease linearly as  increases, showing a similar slope. It thus indicates that there is also a linear correlation between the tunneling current and the photon emission intensity, which agrees with our previous report.^16^

### Excited Plasmon Energy: Atomic Polarizability Dependency

**Figure 5:**
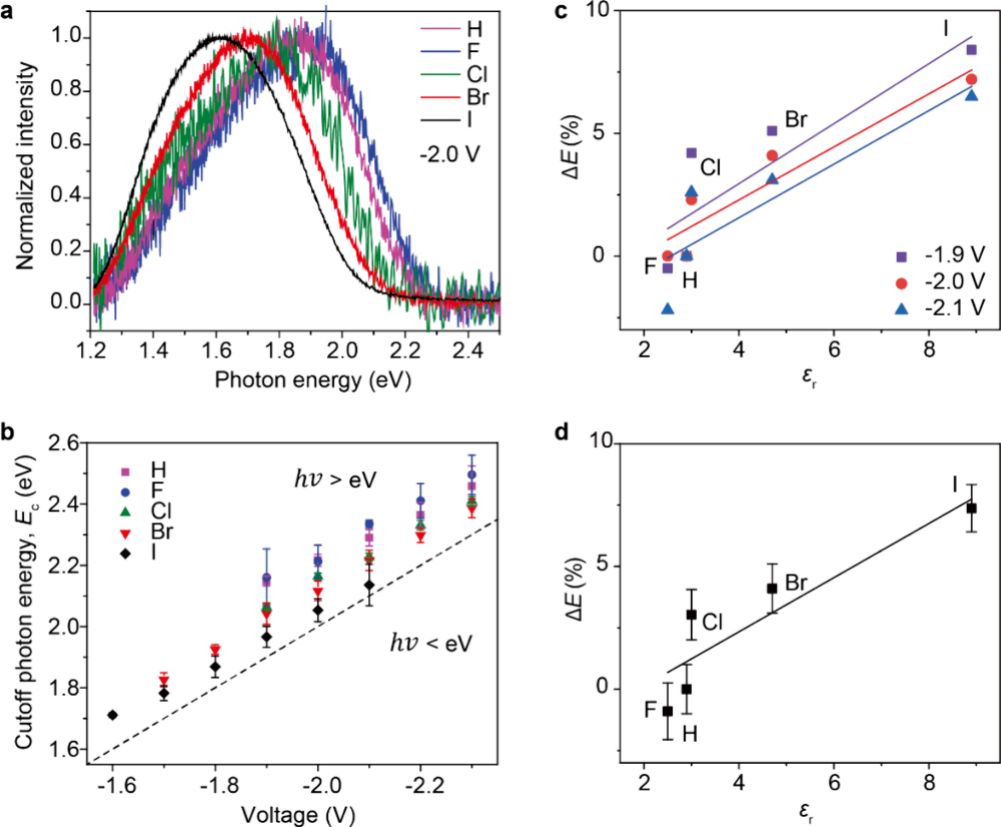
(a) Light emission spectra recorded at −2.0 V bias on Pt substrates with different SAMs. (b) Plot of *E*_c_ as a function of voltage with SC_12_X STJs. The dashed line in b indicates the quantum cutoff with *hv* = *eV*. (c, d) The correlation between the reduced photon energy Δ*E* and ε_*r*_ at different biases (c) and the average from the three biases (d), with error bars from the standard deviation. The lines are linear fits to data. These correlations confirm that α of the SAM terminal atoms changes the electrostatic potential profile of the junctions, tuning the excited plasmon energy.

To measure the excited plasmon energy, we also recorded the light emission spectra to determine how *E*_c_ changes as a function of X (see Methods in the Supporting Information). Figure 5a shows the representative spectra of the emitted photons at an applied bias of −2.0 V and that the spectra red shift from F or H to I by ∼0.2 eV. We note that our junctions consist of planar electrodes (and not of plasmonic resonators or antennas^11,15,18^); therefore, the red shift is not related to the electrode geometry, but it is a molecular effect. This observation confirms our hypothesis that emitted plasmon energy depends on the shape of the molecular tunnel barrier and the resulting electrostatic potential profile across the junction (see Figure 1c) since the other parameters of the tunneling barrier were left unchanged.

By recording *E*_c_ as a function of applied bias (Figure 5b), we observe an over bias emission (*hv* > *eV*) across the measured bias range for all five molecules, which we explain as follows. As the same metal electrodes (Pt and EGaIn) were used for the five molecules, the red-shift of the plasmon spectra in Figure 5a,b should be a molecular effect arising from the difference in X, i.e., the SAM//EGaIn interface. To correlate this red-shift to the electrostatic potential drop at the interface induced by α of the terminal atom, we determined the reduced photon energy Δ*E* (%) relative to X = H with eq 3:
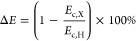
3where Δ*E* is defined as the percentage of the change in photon energy at each voltage. Table 2 summarizes *E*_c_ and Δ*E* at each voltage and shows Δ*E* increases in the sequence: I > Br > Cl > F ≈ H. Further, Δ*E* was plotted against ε_*r*_ (Figure 5c,d) which shows a positive correlation. As ε_*r*_ is directly related to α, these results prove that α can induce a red-shift in the emission photon energy by modifying the electrostatic potential profile of the STJ.

**Table 2:** *E*_c_ and Δ*E* with Different X

	*E*_c_ (eV)			Δ*E* (%)		
terminal atom	–1.9 V	–2.0 V	–2.1 V	–1.9 V	–2.0 V	–2.1 V
H	2.15	2.21	2.29	0	0	0
F	2.16	2.21	2.34	–0.5	0	–2.2
Cl	2.06	2.16	2.23	4.2	2.3	2.6
Br	2.04	2.12	2.22	5.1	4.1	3.1
I	1.97	2.05	2.14	8.4	7.2	6.5

The decrease of overbias emission going from X = F to X = I can be explained as the interface dipoles play a significant role for X = F but are screened for X = I. The polarizability α indicates the ability of an atom or molecule to respond to changes in an electric field by forming induced dipoles. More specifically, the electron clouds of large atoms are distorted more in an external electric field than in small atoms, leading to a dipole. In our work, as X goes from F to I, the value of α increases leading to increasingly large dipoles in applied electric fields. This screening of the electric field by increasingly large halogen atoms leads to an increasingly large potential drop at the SAM//EGaIn interface, which effectively reduces the voltage, *V*_eff_, and decreases the excited plasmon energy. In other words, for monolayers with X = F, the aligned interfaces dipoles with the electric field of the applied bias results in a larger plasmon excitation energy than expected from the applied bias alone leading to overbias emission. In contrast, for monolayers with X = I that can screen, the effect of dipoles will lead to emission close to the quantum cutoff values. Below we carry out additional experiments to test this hypothesis in more detail by systematically varying μ_bond_.

**Figure 6:**
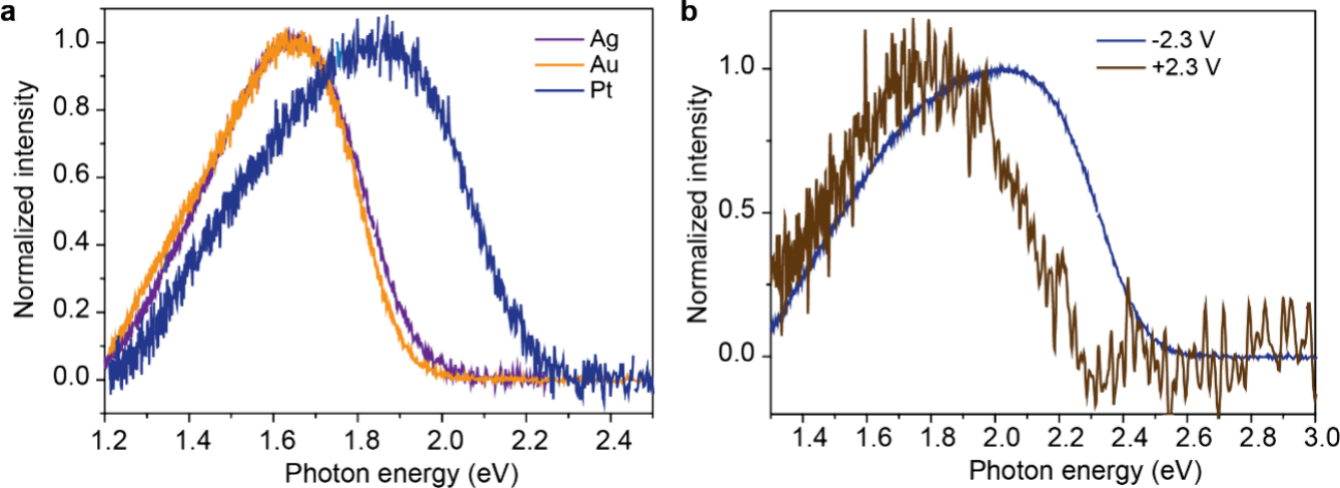
(a) Light emission spectra recorded on different bottom electrodes (Au, Ag, and Pt) at −2.0 V bias with SC_12_H junctions. (b) Emission spectra of SC_12_H junction on Pt electrode collected at ±2.3 V.

In both Figure 5 and Table 2, we observed that *E*_c_ > *eV*. To make sure we are far away from the quantum point contact regime which could result in above quantum threshold photon emission, an estimation was carried out below. Using SC_12_H SAM as an example, the current of the STJ at −1.8 V is ∼100 nA (Figure 3), corresponding to junction conductance *G*_J_ of 7.2 × 10^–4^*G*_0_. By further considering the number of light emission spots of 30 (Figure 4, *V* = −1.8 V) in the junction, the conductance under each light emitting spot is . In case of the SC_12_I SAM, *G*_S_ increases by 2 orders of magnitude, and that is 10^–3^*G*_0_. Based on the above estimation, we can safely conclude that the models involving electron–electron interactions (in the quantum conductance regime) do not apply to our STJs. The model of Fermi level distribution can also be ruled out, as it cannot explain the substrate and bias polarity effect we observed below (Figure 6).

### Excited Plasmon Energy: Interface Dipole Dependency

Figure 2 shows a large difference in ΔΦ among different metal substrates, induced by the different Φ_M_ values and hence the different interface dipoles. As mentioned above, the offset between *E*_c_ and *eV* in our STJs should originate from the dipoles at the molecule–electrode interface, which add to or reduce the effective electric field under externally applied bias. To determine how the μ_bond_ component of the interface dipole affects the excited plasmon energy, we recorded the light emission spectra of SC_12_H junctions on Ag, Au, and Pt substrates at −2.0 V bias. Figure 6a shows that the spectra recorded from the Ag and Au junctions show *E*_c_ ≈ *eV*, while that recorded from Pt gives an ∼0.2 eV blue-shift resulting in *E*_c_ > *eV*. The offset between *E*_c_ and *eV* qualitatively matches the value of ΔΦ for the three metals (Figure 2) and proves that indeed the interface dipoles and associated built-in potentials at the molecule–electrode interface have an influence on the excited plasmon energy. Since ΔΦ is independent of the applied bias, upon reversal of the bias, we expect a red shift. Figure 6b shows, indeed, an ∼0.1 eV red-shift on Pt at positive bias polarity and now *E*_c_ < *eV* applies. The offsets between *E*_c_ and *eV* are much smaller than the ΔΦ measured by UPS which may be caused by the presence of other interface dipoles at, for instance, the SAM//EGaIn interface^71^ or rounding of the tunneling barriers (as mentioned before, assuming rectangular tunneling barriers is like being too simplistic). Note that we increased the electrical bias to ±2.3 V to record the emission spectra shown in Figure 6b, because the light emission was too weak at smaller applied bias in the positive direction (see Methods in the Supporting Information).

## Conclusions

### Atomic Polarizability Changes the Tunnel Barrier and Electrostatic Potential and Plasmonic Properties of Tunnel Junctions

This atomic control originates from the screening of the electric field in the junction, which, in turn, affects the excited plasmon or photon emission energy: molecules with higher α screen the electric field more efficiently, resulting in a lower tunnel barrier, larger potential drop, and thus lower photon energy, and vice versa for molecules with lower α. This leads to the picture outline in Figure 1b where the monolayer can be seen as a double-barrier defined by the alkyl chain and terminal atom (similarly as has been found for another junction^32^) where the potential drop increases with softness of the terminal atom (Figure 1c).

### New Insights Regarding above and below Threshold Photon Emission

Compared to the literature examples introduced in the Background section (Supporting Information), our junctions are far below the quantum conductance and thus are closer to the molecular junctions investigated by McCreery et al. They claimed that the below threshold photon emission in their systems comes from the electron energy loss during charge hopping along the molecule.^46,47^ While in our case, even though there is no hopping (based on the molecular structure of our SAMs and the absence of a thermally activated component in the charge transport process^43,45^), we have evidence for above and below threshold photon emission depending on the terminal atom and work function (and associated interface dipole; Figure 1d). Since interfacial dipoles are directional, we found above or below threshold emission, depending on the applied bias polarity. These observations can explain above threshold emission without the need for invoking higher nonlinear effects (as found for systems with high (close to quantum conductance) currents).^28,34−42^

### Probing Interface Energetics with Excited Plasmon Energy

We also show that by measuring the excited plasmon energy in these junctions as a function of the metal substrates the interface energetics (at the molecule-bottom electrode interface) can be probed qualitatively. The energy of the excited plasmon or emitted photon depends on the effective potential drop across the junction. The presence of interface dipoles (and Fermi-level pinning) generates built-in electric fields that are aligned with, or against, the externally applied electric field and thus increase or lower the energy available to excite plasmons. Experimentally, we show that, with three different metals, the observed photon energy offset (between *E*_c_ and quantum cutoff) qualitatively match with the work function shift (corresponding to the interface dipoles and built-in electric fields) measured with UPS. Moreover, with the same metal, the change of sign in photon energy offset upon reversing the bias polarity further confirms the role of the built-in fields. Here, we can only be qualitative as UPS only characterized the single interface, i.e., molecule-bottom electrode interface, while we know that, once the junction form, there is another molecule-top electrode interface, i.e., SAM//EGaIn interface; as a result, all the energy levels will realign, which will change the interface energetics. On the other hand, the observed lower photon energy offset compared to work function shift measured by UPS may also be related to the formation of image charges^53−55^ in the metal electrode. For example, in EGaIn junctions, image charge effect leads to a modest (1.5 times) reduction in the HOMO–LUMO gap and associated tunneling barrier heights,^72^ but in single-molecule junctions, image charge effects may be 2–3 times larger.^53^ Such effects could be studied in the future by potentiodynamic approaches, for example, where the spectra of the light emission are systematically studied as a function of applied voltage.

### A Plasmonic Approach to Characterize Molecular Junctions in Operando

Although most research on plasmonic tunnel junctions has optical applications in mind, our results suggest that the plasmonic properties can reveal information on molecular tunneling junctions difficult, or perhaps impossible, to obtain with other techniques, such as potential drop profiles or the effect of interface dipoles on tunneling electronics by examining shifts in photon energies. Therefore, we believe that our findings appeal to both the molecular electronics and plasmonics communities and that our results will stimulate new research directions.

## Supporting Information Available

Descriptions of the synthesis and characterization of the compounds, junction preparation, surface characterization, and additional discussion on electrical and optical characterization (PDF)
