# Supplementary material for: Tuning Overbias Plasmon Energy and Intensity in Molecular Plasmonic Tunneling Junctions by Atomic Polarizability
Source: J Am Chem Soc. 2024 Jun 28;146(31):21642–50. doi: 10.1021/jacs.4c05544 (PMC11311224; doi:10.1021/jacs.4c05544)
Supplement: Supplementary file 1 — ja4c05544_si_001.pdf [file ja4c05544_si_001.pdf]

Supporting Information:

Tuning Overbias Plasmon Energy and Intensity in Molecular Plasmonic Tunneling Junctions by Atomic Polarizability

Wei Du[1,2,†], Xiaoping Chen[1,3,†], Tao Wang[1,2], Qianqi Lin[4], and Christian A. Nijhuis[4,]*

[1]*Department of Chemistry, National University of Singapore, 3 Science Drive 3, 117543 Singapore, Singapore*

[2]*Present address: Institute of Functional Nano & Soft Materials (FUNSOM), Soochow University, Suzhou 215123 Jiangsu, P. R. China*

[3]*Department of Chemistry and Environment Science, Fujian Province Key Laboratory of Modern Analytical Science and Separation Technology, Minnan Normal University, Zhangzhou 363000, China*

[4]*Hybrid Materials for Opto-Electronics Group, Department of Molecules and Materials, MESA+ Institute for Nanotechnology, Molecules Center and Center for Brain-Inspired Nano Systems, Faculty of Science and Technology, University of Twente, 7500AE Enschede, the Netherlands*

[†]*These authors contributed equally to this work.*

**Correspondence to e-mail: c.a.nijhuis@utwente.nl*

Table of Contents

1. Background 3

1.1 Tunneling barrier 3

1.2 Electrostatic potential profile, and screening of electric fields 3

1.3 Interface energetics 4

1.4 Mechanisms of plasmon excitation by tunneling electrons 5

1.5 Above and below quantum threshold photon emission 5

2. Method 6

2.1 Synthesis of the molecules. 6

Figure S1. 6

2.2 Junction preparation 11

Figure S2. 12

2.3 Electrical and optical characterizations 12

Figure S3. 13

Figure S4. 14

2.4 XPS characterization 14

Figure S5. 15

Figure S6. 16

Figure S7. 16

2.5 UPS characterization 17

Figure S8. 17

Figure S9. 18

Figure S10. 18

1. Background

1.1 Tunneling barrier

The mechanism of charge transport across molecular junctions is dominated by through molecular bond tunneling where the tunnel barrier width d (in nm) equals the thickness of the SAMs. The tunnel barrier height φ (in eV) is defined as the energy offset between the Fermi level of the electrode and the molecular frontier orbital involved in the charge transport (the highest occupied molecular orbital, HOMO, or the lowest unoccupied molecular orbital, LUMO). Often, the shape of the tunneling barrier is assumed to be rectangular and the potential drops linearly along the molecule and eq. S1 (the simplified Simmons equation) is used

$$J = J_0 e^{-\beta d} \text{ with } \beta = 2\sqrt{2m\varphi/\hbar^2} \tag{S1}$$

where J is the current density (in A cm^{-2}), J_0 is a pre-exponential factor (in A cm^{-2}), β is the tunneling decay coefficient (in $Å^{-1}$), ħ is the reduced Planck's constant and m is the effective mass of the electron. The value of β can be experimentally derived by varying the value of d that is frequently used as an indicator for the quality of the molecular junctions. For junctions with n-alkanethiolate (SC_n) SAMs, the empirical consensus value of β is close to 1.0 n^{-1} (or 0.80 $Å^{-1}$).[1-6] Eq. S1, however, ignores many aspects of the tunneling junctions, and here we only use it to show how J, d, and φ, relate to one and the other.

1.2 Electrostatic potential profile, and screening of electric fields

In principle, in tunnel junctions, the potential drop from the applied voltage can occur at the molecule-electrode interfaces, linearly across the molecule, or, in between these two extremes, *i.e.*, the potential drop in a sigmoidal shape. The shape of the electrostatic potential profile across molecular tunnel junctions has been studied theoretically,[7-9] but experimental investigations are very rare as it is currently not possible to measure the shape of the tunneling barrier directly. Nitzan *et al.*[7] modeled molecular junctions where the molecules were treated

as a cylinder sandwiched between two perfect conductors. They calculated the shape of the electrostatic potential profile as function of the screening length λ of molecular length L and diameter σ and showed that the potential drop along the molecule can be highly non-linear for realistic parameters. The value of λ of CH_2 units has been experimentally determined as 1.5 Å by us,[10,11] in agreement with the calculations by Nitzan *et al.*, resulting in a sigmoidal potential profile.

1.3 Interface energetics

Interface dipoles[12] and built-in fields[13] are always present at interfaces which can significantly alter the potential profile and effective electric field across junctions. The work function Φ of a metal is defined as the energy required to free an electron from Fermi level, E_F, to the vacuum, E_{vac}. At temperatures above 0 K, there is always a probability for electrons to leak out from the metal surface resulting in an interface dipole which changes the work function.[12] When molecules are brought into contact with the metal surface (*via* a chemical bond or physisorption), the electrons leaking out from the metal can be "pushed back" (pillow effect) resulting in a dipole along the interfacial metal-molecule bond, *i.e.* the interface dipole μ_{bond}. The molecular dipole μ_{mol} also contributes to the energy level alignment and alters the observed Φ.[14-23] The change in work function $\Delta\Phi$ due to SAM modification is given by eq. S2:

$$\Delta\Phi = \frac{eN}{\varepsilon_0 \varepsilon_r}(\mu_{bond} + \mu_{mol,\perp}) \qquad (2)$$

where e is the elementary charge (1.602×10^{-19} C), N is the dipole density (here it corresponds to the Γ_{SAM}, in nmol·cm^{-2}), μ_{bond} and $\mu_{mol,\perp}$ is the bond and molecular dipole moment (along surface normal direction, in D), ε_0 is the vacuum permittivity (8.85×10^{-12} F/m), and ε_r *is* the relative dielectric constant.

1.4 Mechanisms of plasmon excitation by tunneling electrons

The exact mechanism of plasmon excitation in tunnel junctions is still not clear[24] and may involve excitation via inelastic tunneling or hot electrons (quantum shot noise[25-27] is important for highly conductive junctions close to the quantum conductance which is not relevant here). Both mechanisms consider plasmon excitation from an energy point of view. For inelastic tunneling excitation, the tunneling charge carrier loses energy and excites plasmons inside the tunneling barrier, while in the hot electron picture the charge carrier first tunnels across the barrier elastically and then excites plasmons. According to theoretical calculations, inelastic tunneling excitation of surface plasmons is more efficient (by several orders of magnitude) than hot electron excitation.[28] The reason behind this can be understood as the competition between plasmon excitation and electron-hole pair excitation. The latter one can be considered as a "quenching" process which happens with a larger probability in the metal electrode close to the electron bath than in the tunneling barrier.[24] Experimental results of tunnel junctions having asymmetric metal electrodes also show similar emission spectra at two bias polarities.[29] This would not happen in the case of hot electron excitation in which the plasmon property should depend on the counter electrode material. Thus, inelastic tunneling excitation is generally considered as the dominant mechanism for plasmon excitation in tunnel junctions by the community.

1.5 Above and below quantum threshold photon emission

An offset between E_c and eV is frequently observed, resulting in above and below quantum threshold photon emission. Small offsets (<100 meV) above the quantum threshold have been understood as the result of the energy distributions of the injected electrons at finite electron temperature (up to 1000 K).[30,31] Larger offsets (up to 2 eV or even 3 eV) above the quantum threshold were explained by quantum shot noise models[25-27], hot carrier based

emission with elevated electronic temperature[32-35] (>1000 K), or multi-electron process involving electron-electron (or electron-plasmon) interactions.[36,37] The different models are to some extent related with each other and even co-existent in the same junction.[35,38] Usually, as the tunnel current increases, there is larger chance for multi-electron process and hot carrier based emission, leading to more obvious above threshold emission. On the other hand, offsets below the quantum threshold are considered to involve energy loss step due to the interband transition of the metal electrode (e.g., the interband transition between *sp* and *d* bands of gold which happens at the energy range of 2.6–2.8 eV[39]) or electron energy loss during the charge transport[40,41] (corresponding to the hopping mechanism).

2. Method

2.1 Synthesis of the molecules.

The synthesis route of the $X(CH_2)_{12}SH$ is similar to the published paper[42] by Wang *et al.* following the below scheme shown in Fig. S1.

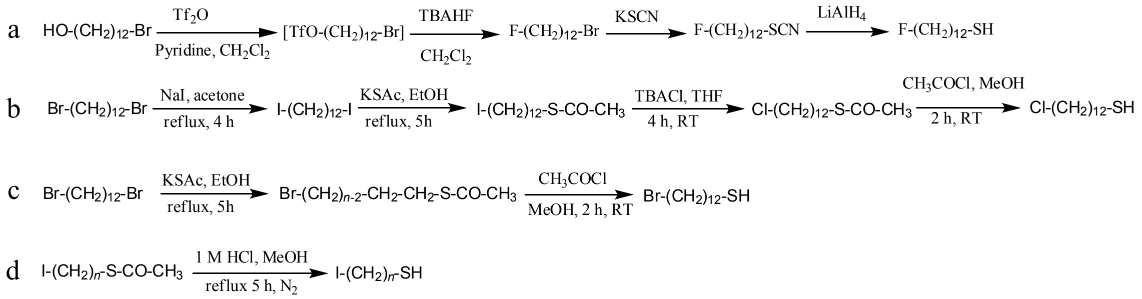

Figure S1. Synthesis scheme of the $X(CH_2)_{12}SH$ molecules.

All the chemicals and regents were ordered from Sigma-Aldrich, Tokyo Chemical Industry CO., Ltd, or Alfa Aesar without further purification. Elga Purelab option-Q system was used to produce deionized water. The silica gel (60 Å/40-63 μm) used for column chromatography was purchased from Sigma-Aldrich. ^1H and ^{13}C NMR spectra were collected

from Bruker Avance 300 MHz spectrometer or Bruker Avance 400 MHz spectrometer using $CDCl_3$ as solvent. Electron ionization (EI) mass spectra were analyzed from Finnigan LCQ mass spectrometer.

1-bromo-12-fluorododecane. The 12-fluorododecane-1-thiol was synthesized following Figure S1a.[43] To a two-neck round bottom flask, we added 12-bromo-1-dodecanol (5.00 g, 18.8 mmol), 100 mL distilled DCM, and pyridine (1.74 mL, 21.6 mmol) under N_2 and cooled down the solution with salty ice water, followed by dropwise addition of trifluoromethanesulfonic anhydride, Tf_2O (6.09 g, 21.6 mmol). The mixture was allowed to stir for 30 min and then washed with 1M HCl solution. The organic DCM layer was separated and dried with anhydrous Na_2SO_4. 9.17 mL (22 mmol) tetrabutylammonium hydrogen difluoride (TBAHF) was added to the filtrate and stirred for another 2 h before the solvent was removed. 100 mL hexane was poured into the residue flask and stirred for 3 h to extract the raw product. This step was repeated three times and the hexane solution was collected together and concentrated. The raw product was purified by column chromatography with hexane as eluent and collected at the second section with a yield of 45 % (2.27 g). ^{1}H-NMR (400 MHz, $CDCl_3$): δ 1.28-1.40 (m, 16H, -$(CH_2)_8$-CH_2CH_2F), 1.65-1.71(m, 2H, -CH_2-CH_2F), 1.85 (p, J =6.8 Hz, 2H, -CH_2-CH_2Br), 3.40 (t, J =6.8 Hz, 2H, -CH_2-Br), 4.42 (dt, J_{HF} =47.6 Hz, J_{HH} =6.4 Hz, 2H, -CH_2-F) ppm. ^{13}C-NMR (100 MHz, $CDCl_3$): δ = 25.28 (d, $^3J_{CF}$ = 5.3 Hz, -*C*-C-C-F), 28.30, 28.89, 29.36, 29.54, 29.61, 30.55 (d, $^2J_{CF}$ = 19.1 Hz, -*C*-C-F), 32.98, 34.08, 84.30 (d, $^1J_{CF}$ = 163.0 Hz, -*C*-F) ppm. EI-MS: 268 (M^+).

1-fluoro-12-thiocyanatododecane. 1-fluoro-12-thiocyanatododecane was synthesized following the reported literature.[44] We added potassium thiocyanate (0.50 g, 5.16 mmol) to a two-neck bottom flask followed by 20 mL ethanol. The mixture was heated up to reflux. 1-bromo-12-fluorododecane (0.88 g, 3.30 mmol) in 10 mL ethanol was added dropwise in 20 min. The mixture was refluxed for 3 h before cooling down to room temperature. The solvent

was removed and the residue was dissolved in DCM and washed with DI water for three times. The organic layers were combined, dried over anhydrous Na_2SO_4, concentrated. The crude product was purified by column chromatography with ethyl acetate: hexane = 1:20. Yield 75 % (0.60 g). ^{1}H-NMR (400 MHz, $CDCl_3$): δ 1.27-1.42 (m, 16H, -$(CH_2)_8$-CH_2CH_2F), 1.64-1.70 (m, 2H, -CH_2-CH_2F), 1.81 (p, J =7.2 Hz, 2H, -CH_2-CH_2SCN), 2.93 (t, J =7.2 Hz, 2H, -CH_2-SCN), 4.42 (dt, J_{HF} =47.6 Hz, J_{HH} =6.4 Hz, 2H, -CH_2-F) ppm. ^{13}C-NMR (100 MHz, $CDCl_3$): δ = 25.22 (d, $^3J_{CF}$ = 5.6 Hz, -*C*-C-C-F), 28.01, 28.93, 29.28, 29.39, 29.51, 29.53, 29.95, 30.48 (d, $^2J_{CF}$ = 19.2 Hz, -*C*-C-F), 34.15, 84.24 (d, $^1J_{CF}$ = 163.1 Hz, -*C*-F), 112.43 (-S-*C*≡N) ppm. EI-MS: 244 (M^+).

12-fluorododecane-1-thiol. We used lithium ammonium anhydride to convert the SCN to SH.[44] We cooled down a two-neck flask with ice water under N_2, then added 20 mL distilled diethyl ether and lithium ammonium anhydride (0.25 g, 7.41 mmol). To the mixture, 1-fluoro-12-thiocyanatododecane (0.50 g, 2.05 mmol) in 5 mL distilled diethyl ether was added dropwise. The mixture was allowed to stir for another 10 min before degassed DI water was added to quench the reaction. 3 M HCl was added to the solution until the solution was clear. The mixture was extract with diethyl ether for three times and the organic layer was combined and washed with DI water for three times. The organic layer was then dried, concentrated, and finally purified with column chromatography with hexane as eluent, yielding 0.40 g (89 %) 12-fluorododecane-1-thiol. ^{1}H-NMR (400 MHz, $CDCl_3$): δ 1.27-1.39 (m, 16H, -$(CH_2)_8$-CH_2CH_2F), 1.61-1.72 (m, 4H, -CH_2-CH_2F and -CH_2-CH_2SH), 2.52 (q, J =7.2 Hz, 2H, -CH_2-SH), 4.44 (dt, J_{HF} =47.6 Hz, J_{HH} =6.4 Hz, 2H, -CH_2-F) ppm. ^{13}C-NMR (75 MHz, $CDCl_3$): δ = 24.77, 25.29 (d, $^3J_{CF}$ = 5.5 Hz, -*C*-C-C-F), 28.51, 29.20, 29.37, 29.63, 29.66, 30.56 (d, $^2J_{CF}$ = 19.2 Hz, -*C*-C-F), 34.18, 84.32 (d, $^1J_{CF}$ = 163.0 Hz, -*C*-F) ppm. EI-MS: 220 (M^+). Melting point: -1.8 °C.

1,12-diiodododecane. 6.20 g (18.9 mmol) 1,12-dibromododecane and 9.60 g (64.1 mmol) sodium iodine were added to a flask with 150 mL acetone and refluxed for 5 h. The solvent was removed under reduced pressure. DCM was added to dissolve the residue. The DCM solution was washed with DI water 3 times, dried over anhydrous Na_2SO_4 and then concentrated. The concentrated solid was used without further purification with a yield of 95 % (7.60 g, 18.0 mmol). ^{1}H-NMR (300 MHz, $CDCl_3$): δ 1.27-1.39 (m, 16H, -$(CH_2)_8$-CH_2CH_2I), 1.81 (p, J =7.2 Hz, 4H, -CH_2-CH_2I), 3.18 (t, J =7.2 Hz, 4H, -CH_2-I) ppm. ^{13}C-NMR (75 MHz, $CDCl_3$): δ =7.44 (-*C*-I), 28.69, 29.55, 29.67, 29.72, 30.66, 33.73 ppm. EI-MS: 422 (M^+).

12-iodo-1-dodecanethioacetate. 5.00 g (11.8 mmol) 1,12-diiodododecane was dissolved with 200 mL acetonitrile and refluxed. 1.35 g (11.8 mmol) potassium thioacetate was separated into three portions and added at intervals of 1.5 hours. The solution was then cooled down, and evaporated under reduced pressure. The residue was dissolved with DCM and washed with DI water, dried over anhydrous Na_2SO_4 and concentrated. The crude product was purified over hexane and DCM at a volume ratio of 10:1on column chromatography, yielding 35 % (1.53 g, 4.13 mmol). ^{1}H-NMR (300 MHz, $CDCl_3$): δ 1.25-1.36 (m, 16H, -$(CH_2)_8$-CH_2CH_2I), 1.55 (p, J =7.2 Hz, 2H, -CH_2-CH_2S), 1.81 (p, J =7.2 Hz, 2H, -CH_2-CH_2I), 2.31 (s, 3H, -SCOCH_3), 2.85 (t, J =7.2 Hz, 2H, -CH_2-S), 3.17 (t, J =7.2 Hz, 2H, -CH_2-I) ppm. ^{13}C-NMR (75 MHz, $CDCl_3$): δ =7.39 (-*C*-I), 28.64, 28.92, 29.20, 29.27, 29.50, 29.54, 29.60, 30.61, 30.76, 33.68, 196.09 (-S-*C*O-) ppm. EI-MS: *m/z* 369.9 (M^+).

12-chloro-1-dodecanethioacetate. We used reported method to convert iodo to chloro.[45] 12-iodo-1-dodecanethioacetate (1.00 g, 2.70 mmol) and 3.20 g (11.51 mmol) tetrabutylammonium chloride (TBACl), 100 mL tetrahydrofuran (THF) were added into a two-neck flask and stirred for 4 h under room temperature. THF was then removed and the residue was dissolved with DCM and washed with water, dried, concentrated, and purified with hexane: ethyl acetate = 10:1. Yield 95 % (0.71g, 2.56 mmol). ^{1}H-NMR (300 MHz, $CDCl_3$): δ 1.26-1.42

(m, 16H, -($CH_2)_8$-CH_2CH_2Cl), 1.55 (p, *J* =7.2 Hz, 2H, -CH_2-CH_2S), 1.76 (p, *J* =6.9 Hz, 2H, -CH_2-CH_2Cl), 2.31 (s, 3H, -SCOCH_3), 2.85 (t, *J* =7.2 Hz, 2H, -CH_2-S), 3.52 (t, *J* =6.9 Hz, 2H, -CH_2-Cl) ppm. ^{13}C-NMR (75 MHz, $CDCl_3$): δ =27.01, 28.93, 29.00, 29.22, 29.27, 29.56, 29.62, 30.74, 32.78, 45.28 (-*C*-Cl), 196.12 (-S-*C*) ppm. EI-MS: *m/z* 278.1 (M^+).

12-chloro-1-dodecanethiol. 12-chloro-1-dodecanethioacetate (0.50 g, 1.80 mmol) and 20 mL methanol were added into a two-neck flask under N_2. The mixture was degassed for 20 min and cooled down with ice water. 2 mL acetyl chloride was added dropwise into the mixture. The mixture was allowed to stir for 2 h before quenched by DI water. The organic compound was extracted with DCM and washed with DI water for three times. After drying, concentration and purification (hexane as solvent), 0.36 g (yield 85 %) product was obtained. ^{1}H-NMR (300 MHz, $CDCl_3$): δ 1.26-1.38 (m, 16H, -$(CH_2)_8$-CH_2CH_2Cl), 1.59 (p, *J* =7.2 Hz, 2H, -CH_2-CH_2SH), 1.75 (p, *J* =6.9 Hz, 2H, -CH_2-CH_2Cl), 2.51 (q, *J* =7.2 Hz, 2H, -CH_2-SH), 3.51 (t, *J* =6.9 Hz, 2H, -CH_2-Cl) ppm. ^{13}C-NMR (75 MHz, $CDCl_3$): δ = 24.73 (-*C*-SH), 26.97, 28.46, 28.97, 29.15, 29.53, 29.58, 29.61, 32.75, 34.14, 45.22 (-*C*-Cl) ppm. EI-MS: 236 (M^+). Melting point: 21.5 °C.

12-bromo-1-dodecanethioacetate. 12-bromo-1-dodecanethioacetate was obtained using the same method as 12-iodo-1-dodecanethioacetate. ^{1}H-NMR (300 MHz, $CDCl_3$): δ 1.26-1.42 (m, 16H, -$(CH_2)_8$-CH_2 CH_2Br), 1.56 (p, *J* = 7.2 Hz, 2H, -CH_2-CH_2-S), 1.85 (p, *J*= 6.9 Hz, 2H, -CH_2-CH_2-Br), 2.32 (s, 3H, -CH_2-SCOCH_3), 2.86 (t, *J* = 7.2 Hz, 2H, -CH_2-$SCOCH_3$), 3.40 (t, *J* = 6.9 Hz, 2H, -CH_2-Br) ppm. ^{13}C-NMR (75 MHz, $CDCl_3$): δ = 28.31, 28.89, 28.95, 29.23, 29.30, 29.55, 29.57, 29.63, 30.77, 32.98, 34.15, 196.17 (*C*=O) ppm. EI-MS: 322 (M^+).

12-bromo-1-dodecanethiol. 12-bromo-1-dodecanethiol was synthesized using the same method as 12-chloro-1-dodecanethiol. ^{1}H-NMR (300 MHz, $CDCl_3$): δ 1.27-1.39 (m, 16H, -$(CH_2)_8$-CH_2CH_2Br), 1.61 (p, *J* =7.2 Hz, 2H, -CH_2-CH_2SH), 1.85 (p, *J* =6.9 Hz, 2H, -CH_2-CH_2Br), 2.52 (q, *J* =7.2 Hz, 2H, -CH_2-SH), 3.40 (t, *J* =6.9 Hz, 2H, -CH_2-Br) ppm. ^{13}C-NMR

(75 MHz, $CDCl_3$): δ = 24.79 (-*C*-SH), 28.32, 28.51, 28.90, 29.20, 29.55, 29.63, 29.65, 32.99 (-*C*-Br), 34.14, 34.18 ppm. EI-MS: 280 (M^+). Melting point: 28.8 ℃.

12-iodo-1-dodecanethiol. The synthesis of 12-iodo-1-dodecanethiol[45] was slightly different from the 12-bromo-1-dodecanethiol. We added 12-iodo-1-dodecanethioacetate (1.00 g, 2.7 mmol), 50 mL methanol into a two-neck flask under N_2 and degassed the mixture for 20min before refluxing for 5 h. After the mixture was cooled down to room temperature, the methanol was removed by rotary evaporator. The residue was picked up by DCM and washed with DI water. After workup, the raw product was purified on column using hexane. Yield 20 % (0.18g, 0.54 mmol). ^{1}H-NMR (300 MHz, $CDCl_3$): δ 1.27-1.37 (m, 16H, -$(CH_2)_8$-CH_2CH_2I), 1.60 (p, J =7.2 Hz, 2H, -CH_2-CH_2SH), 1.81 (p, J =6.9 Hz, 2H, -CH_2-CH_2I), 2.51 (q, J =7.2 Hz, 2H, -CH_2-SH), 3.18 (t, J =6.9 Hz, 2H, -CH_2-I) ppm. ^{13}C-NMR (75 MHz, $CDCl_3$): δ =7.40 (-*C*-I), 24.79, 28.51, 28.67, 29.20, 29.53, 29.62, 30.64, 33.71, 34.18 ppm. EI-MS: 328 (M^+). Melting point: 28.2 ℃.

2.2 Junction preparation

We prepared the three metal bottom electrodes with the previously reported procedure of template stripping. The thickness of the metal is 20 nm to ensure the transmission of light. Figure S2 shows the AFM images of the template stripped (TS) Au, Ag, and Pt surfaces. The typical root-mean-square (rms) surface roughness prepared by the TS method is 0.2±0.1 nm for the Pt, 0.3±0.1 nm for the Au, and 1.2±0.2 nm for the Ag substrates (all measured over an area of 1 μm × 1 μm). The size of the grains also follows the trend of Pt < Au < Ag. To form the SAMs, we immersed the fresh TS surfaces in 3 mM ethanolic solution containing the desired thiol molecules for 3 h under nitrogen environment. After the growth of SAMs, we washed the substrates with ethanol (to remove physisorption) and blow with nitrogen.

The SC_nX SAMs on Au and Ag with X = H have been thoroughly studied in the past.[1-6] These SAMs on Pt have similar packing densities and thickness as SAMs on Ag and Au.[46] The SAMs on Pt with X = F, Cl, Br, or I, were characterized with X-ray photoelectron spectroscopy (XPS) from which we determined the relative surface coverage Γ_{SAM} (nmol cm^{-2}) using $SC_{12}Br$ SAM on Pt as a reference and the results are listed in Table 1. The values of Γ_{SAM} are within error indistinguishable from which we conclude that all SAMs have a similar structure.

The junction was formed by stamping the EGaIn top electrode (held in PDMS device) on top of the SAM modified bottom electrodes.

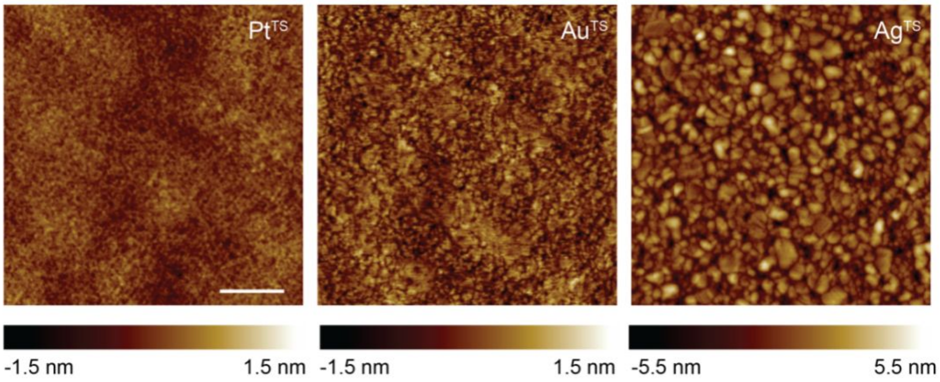

Figure S2. AFM images of the template stripped surfaces of Au (a), Ag (b) and Pt (c).

2.3 Electrical and optical characterizations

For electrical characterization of the STJs, the bias was applied to the STJs following the sequence of 0 → -1.8 → 0 → +1.8 → 0 V for each $J(V)$ trace. Here, we scanned the voltage up to -1.8 V as most of the junctions can withstand an applied bias voltage of -1.8 V, but a fraction of the junctions (40-50%) withstood a somewhat higher electrical biases up to -2.3 V. For each type of SAMs, we measured 50-200 $J(V)$ curves from 5-10 junctions fabricated on 3-4 Pt substrates, based on which we determined the log-average values of the current densities, $\log_{10}|J|$, and the log-standard deviation σ_{log}, as shown in Fig. 3 in the main text.

Figure S3 shows the schematic of the optical characterization. Light emission from the STJs was recorded with an inverted optical microscope (Nikon Eclipse T*i-E*) with bias applied to the EGaIn top electrode (and bottom electrode was grounded). Light emission images shown in Figure 4 were recorded with an electron multiplying CCD (EMCCD) camera (iXon Ultra 897) with 300 EM Gain, using an oil immersion objective (100 ×, numerical aperture NA = 1.49). Integration time of 30 s and 2 min were used depending on the intensity of the light emission with different molecules. We also collected the spectra of the light emission from the STJs using an Andor spectrometer (Shamrock 303i), with 4 conversion Gain. After subtracting the background (measured with the same setting without applying bias), the spectra were normalized and given in Fig. 5 and Fig. 6. Note that the emission spectra in Fig. 6b show weaker light emission at +2.3 V than at -2.3 V. This is due to the lower tunnelling current at high positive bias due to a small rectification effect.[47].

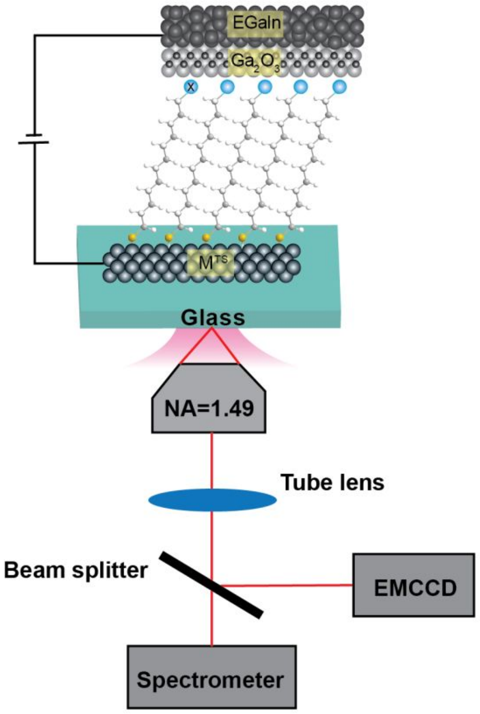

Figure S3. Schematic illustration of the optical characterization.

We used the method shown in Figure S4 to determine E_c from the intersection of the two

red dashed lines, indicated by the red arrow.

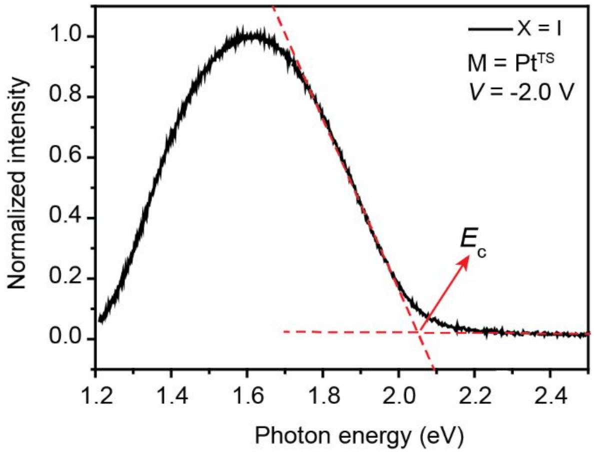

Figure S4. The determination of E_c, using one spectrum recorded at -2.0 V bias with SAMs of $SC_{12}I$ on the Pt substrate as an example.

2.4 XPS characterization

We characterized the freshly prepared SAMs with XPS at the SINS (Surface, Interface and Nanostructure Science) beamline of Singapore Synchrotron Light Source (SSLS) using similar procedures as reported before.[48] The experiments were conducted in the normal emission mode, at room temperature, and in ultra-high vacuum (UHV) chamber (~1×10^{-10} mbar pressure). We calibrated the photo energy using a sputtered gold film as the reference (Au $4f_{7/2}$ core level peak of 84 eV). All the XPS data were fitted using XPS PEAK v. 4.1 (Raymund Kwok, The Chinese University of Hong Kong, Shatin, Hong Kong). The profile of each peak was fitted using a Shirley background and Voigt function (Gaussian : Lorentzian = 7/3). Figure S5 shows the XPS peak signals of C 1*s*, S 2*p*, and the metal (Ag 3*d*, Au 4*f*, Pt 4*f*) with the $SC_{12}H$ SAMs on Ag, Au, and Pt substrates. On Pt substrates, we observed two types of S 2*p* signals (161.9 and 163.1 eV) which are different from the same SAM on Au or Ag substrates. The peak at 161.9 eV constributes to the chemisorbed M-S bonds and the extra component of S 2*p* at 163.1 eV with Pt substrates may be attributed to physisorbed species.[49] Figure S6 shows the XPS peak signals of C 1*s*, S 2*p*,

and Pt 4*f* with the $SC_{12}X$ (X = F, Cl, Br or I) SAMs on Pt substrates. The spectra show two types of C 1*s* signals which are associated with the carbons in the alkyl chain (with lower binding energy of 284.8 eV) and the carbon connected to the halogen (with higher binding energy). For the latter one, as the electronegativity χ of the terminal atom increases, the carbon next to the halogen further shifts to higher binding energy from 287.6 eV for X = F to 285.5 eV for X = I.[49] Figure S7 shows the F 1*s*, Cl 2*p*, Br $3p_{3/2}$, and I 3*d* peak signals with the $SC_{12}X$ SAMs on Pt substrates. Br 3*p* was chosen instead of Br 3*d* because of overlap with Pt 4*f* region. With the above results, we conclude that these thiolates form densely packed SAMs.

For the relative surface coverage of $SC_{12}X$ SAMs on Pt, we compared the ratio of peak areas of Pt 4*f* for each X relative to that of $SC_{12}Br$ SAM (which was found to be 1.1 nmol·cm^{-2} on Ag^{TS} (ref 49)), assuming that the coverage of the overlayer is the only factor altering the intensity of photoelectrons coming from the Pt surfaces.

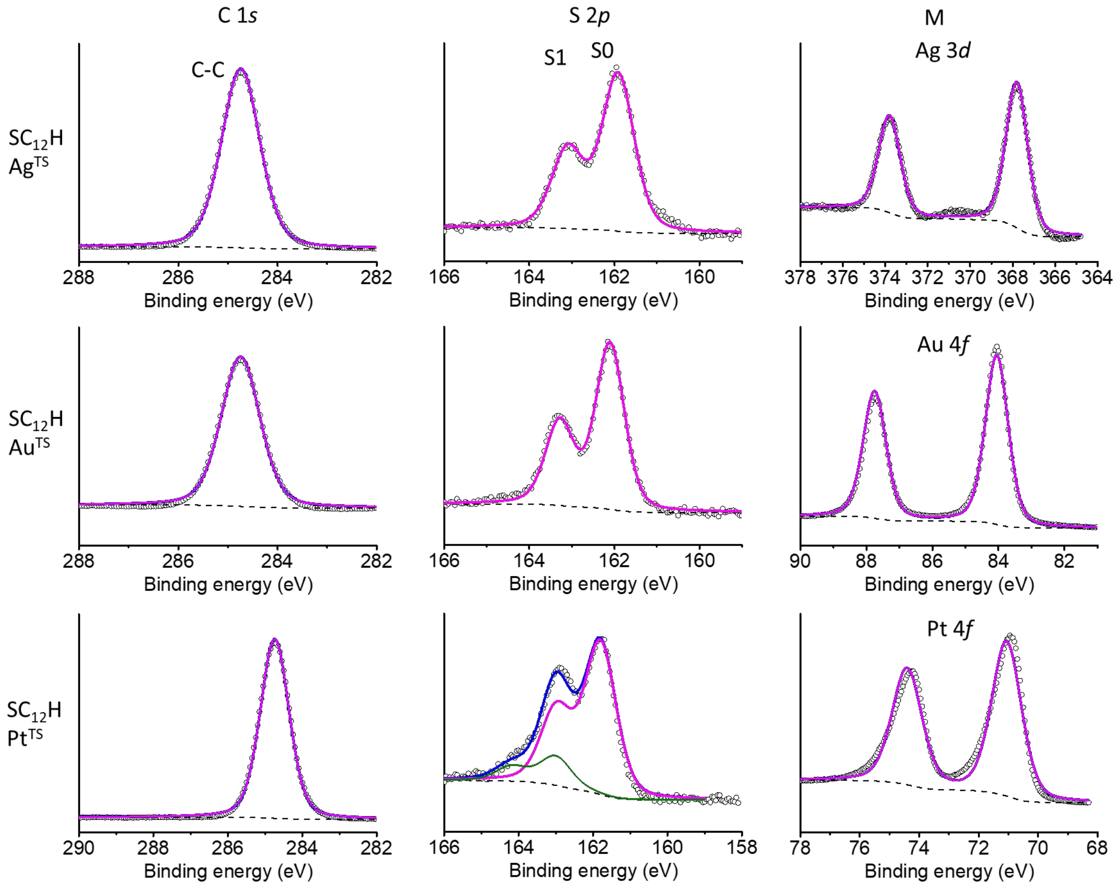

Figure S5. XPS spectra (normal emission) of C 1*s*, S 2*p*, Ag 3*d*, Au 4*f*, and Pt 4*f* of the $SC_{12}H$

SAMs on different metal substrates.

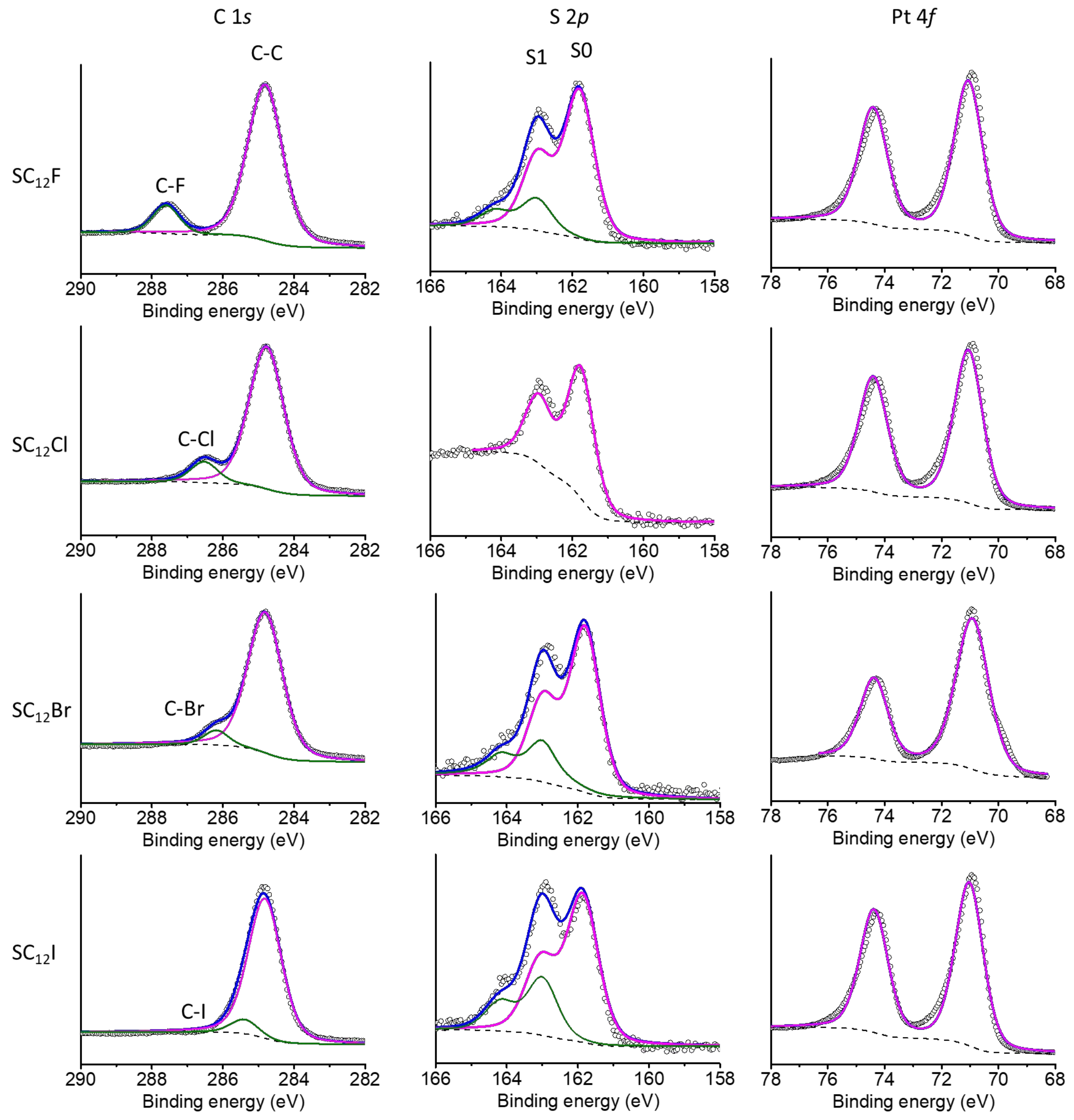

Figure S6. XPS spectra (normal emission) of C 1*s*, S 2*p*, Pt 4*f* with the $SC_{12}X$ SAMs on Pt.

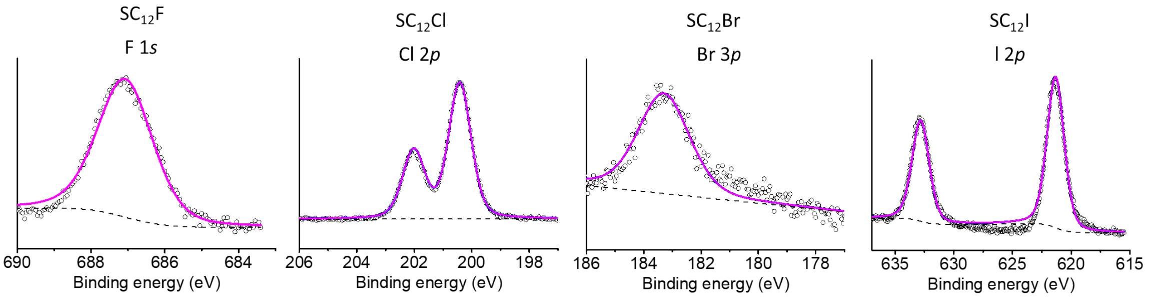

Figure S7. XPS spectra (normal emission) of F 1*s*, Cl 2*p*, Br $3p_{3/2}$, and I 3*d* of the $SC_{12}X$ SAMs

on Pt substrates.

2.5 UPS characterization

We also conducted UPS characterization of these $SC_{12}X$ SAMs to determine the energy level alignment of the SAM-bottom electrode interface. The UPS spectra were measured on SINS beamline. The Fermi edges and work function values were calibrated with a clean gold foil with work function of 5.10 eV. The work function $\Phi_{M\text{-}SAM}$ and HOMO onset were obtained from the secondary electron cut-off (SECO) and the beginning of the valence band, respectively, by linear extrapolation of the lower binding energy side of the HOMO peak and secondary electron cut-off to the base line.[49]

Figure S8 shows the UPS spectra of $SC_{12}H$ SAMs on Au, Ag, and Pt substrates and Fig. S9 shows the UPS spectra of the $SC_{12}X$ (X = F, Cl, Br or I) SAMs on Pt substrates. The values of $\Phi_{M\text{-}SAM}$ and HOMO-onset are summarized in Tables S1-2 and Table 1 in the main text.

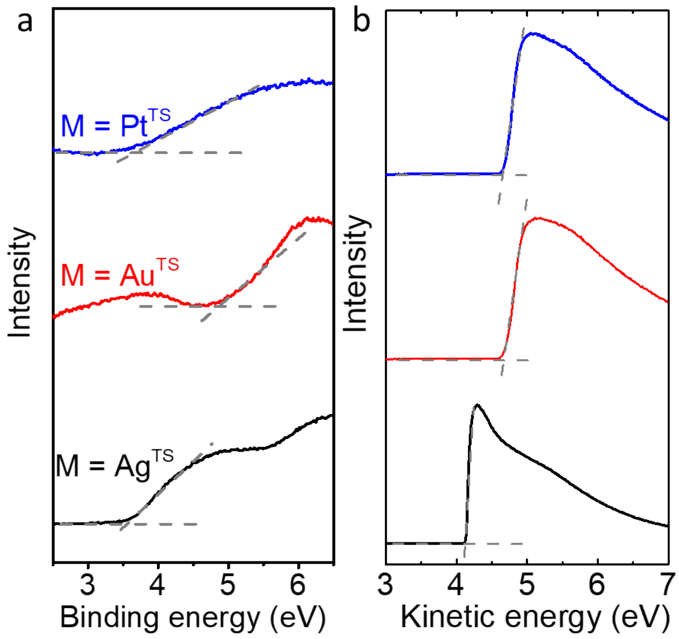

Figure S8. UPS spectra of the $SC_{12}H$ SAMs on Au, Ag, and Pt substrates. The valence band (a) and secondary electron cut-off (b) from which we determined the HOMO-onset values and Φ_{M-}

SAM respectively.

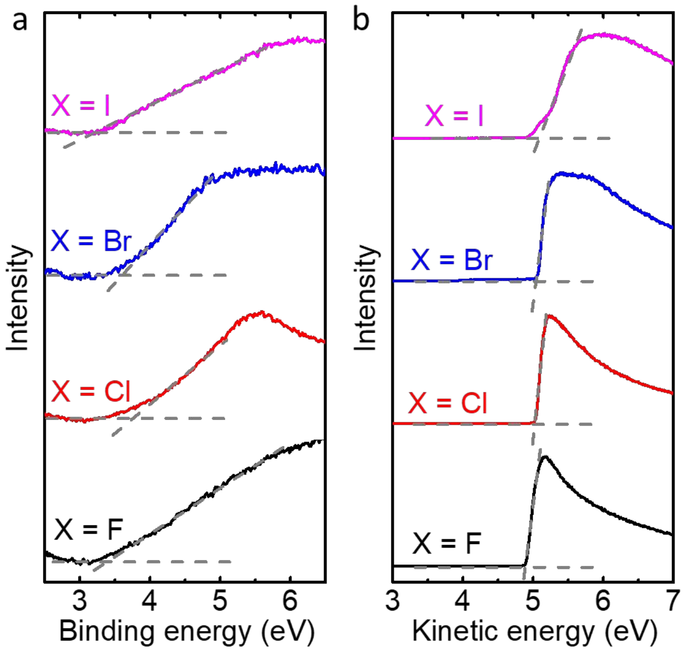

Figure S9. UPS spectra of the $SC_{12}X$ SAMs on Pt substrates. The valence band (a) and secondary electron cut-off (b).

Figure S10 shows the energy level diagram of the Pt-$SC_{12}X$//EGaIn junctions with zero bias, where X = F, Cl, Br or I. Based on the HOMO energy determined from the UPS measurements, and E_g calculated by gas phase density-functional theory (DFT) taken from ref. 49, we have extracted the tunnel barrier height φ for X = F to I (Table 1 in the main text). Here, the tunnelling barrier height is defined as the offset in energy between the LUMO and Fermi-level of the electrode. For X = Cl, Br and I, the LUMO is closer to Fermi energy than the HOMO, making the LUMO the transport orbital. For X = F, based on the values shown in Table 1, HOMO seems to be closer to Fermi energy than LUMO. However, by taking into consideration the image charge effect, which can reduce E_g by a factor of 1.5 in EGaIn junctions due to the energy level renormalization,[50] the LUMO of X = F will be lowered and becomes the transport orbital.

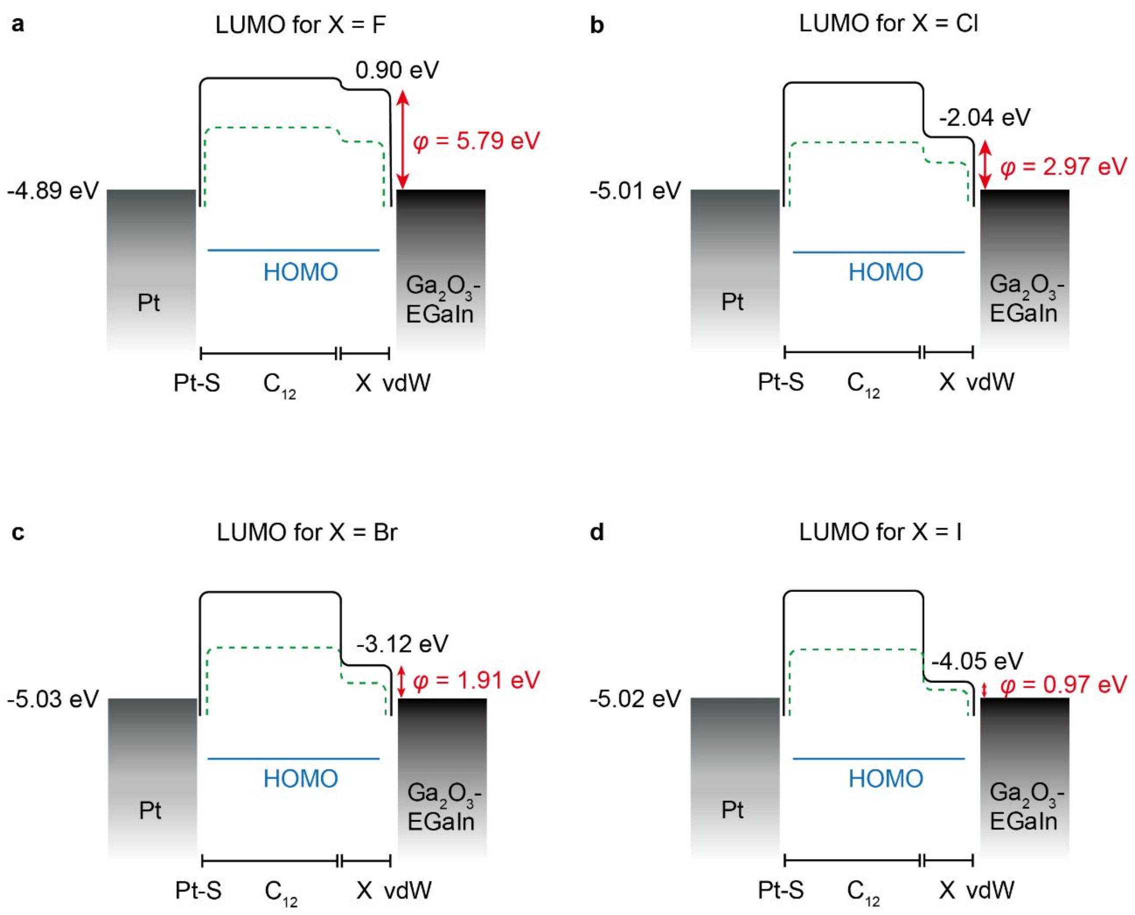

Figure S10. Energy level diagram of the Pt-$SC_{12}X$//EGaIn junctions with zero bias, where X = F, Cl, Br or I. The shapes of the tunnel barriers are indicated by the black solid lines which change as a function of X, the red arrows indicate the barrier height φ defined as the energy offset between LUMO and Fermi energy, and the blue solid lines indicate the HOMO orbitals. All the energy levels are drawn to scale based on the values summarized in Table 1 of the main text. Note that the tunnel barriers are drawn with arbitrarily chosen rounded corners for simplicity. In addition, the green dashed lines schematically illustrate the lowering of the LUMO level and barrier height φ due to the image charge effects as explained briefly in the main text.

With UPS characterization, we could study μ_{bond} and $\mu_{\mathrm{mol},\perp}$ independently following the below procedure. First, we calculated $\Delta\Phi$ from $\Phi_{\mathrm{M\text{-}SAM}}$ and Φ_{M}. Second, we characterized the same $SC_{12}H$ SAM on different metal substrates (Ag, Au and Pt) where μ_{bond} changes as a

function of the metal electrode material. Since $\mu_{\mathrm{mol},\perp}$ (-0.8 D) is known for alkanethiolate SAMs on Au surface,[51] according to the different tilt angles for Au, Ag and Pt surfaces, we can also calculate $\mu_{\mathrm{mol},\perp}$ for alkanethiolate SAMs on Ag and Pt surfaces. Further, referring to eq. S2 and taking Γ_{SAM} of ~1.0 nmol·cm^{-2} and ε_r of 2.9 from previous report,[49] we could calculate μ_{bond} with the three metal substrates (as shown Table S1). Third, we used the same Pt metal substrate but studied different SAMs in the form of $SC_{12}X$ (X = F, Cl, Br, and I) where molecular dipole changes due to the different terminal atom. As μ_{bond} with Pt has been calculated on Table S1, by using eq. S2 again, we can also calculate $\mu_{\mathrm{mol},\perp}$ with different X, which is summarized in Table S2.

Table S1 $SC_{12}H$ SAM on Ag, Au and Pt substrates.

Metal	Φ_M (eV) [a]	$\Phi_{M\text{-}SAM}$ (eV)	$\Delta\Phi$ (eV)	$\mu_{\mathrm{mol},\perp}$ (D)	μ_{bond} (D)
Ag	4.3	4.13	-0.17	-0.91	0.69
Au	5.1	4.68	-0.42	-0.80[b]	0.26
Pt	5.7	4.67	-1.03	-0.92	-0.39

[a]The values of Φ_M are taken from Ref. 52.

[b]The value of $\mu_{\mathrm{mol},\perp}$ on Au surface is taken from Ref. 51. The tilt angles for $SC_{12}H$ SAM on Au, Ag and Pt surfaces are taken as 30°, 10° and 5°, respectively.

Table S2 $SC_{12}X$ (X = F, Cl, Br and I) SAMs on Pt substrates.

X	Φ_M (eV)[a]	$\Phi_{M\text{-}SAM}$ (eV)	$\Delta\Phi$ (eV)	μ_{bond} (D)	$\mu_{\mathrm{mol},\perp}$ (D)
F	5.7	4.89	-0.81	-0.39	-0.50
Cl	5.7	5.01	-0.69	-0.39	-0.52
Br	5.7	5.03	-0.67	-0.39	-1.00
I	5.7	5.02	-0.68	-0.39	-2.27

[a]The values of Φ_M are taken from Ref. 52.

[b]The value of μ_{bond} is taken from the calculation result of Table S1.

References

1. Wan, A.; Jiang, L.; Sangeeth, C. S. S.; Nijhuis, C. A. Reversible soft top-contacts to yield molecular junctions with precise and reproducible electrical characteristics. *Adv. Funct. Mater.* **2014**, 24, 4442–4456.
2. Jiang, L.; Sangeeth, C. S. S.; Wan, A.; Vilan, A.; Nijhuis, C. A. Defect scaling with contact area in EGaIn-based junctions: Impact on quality, Joule heating, and apparent injection current. *J. Phys. Chem. C* **2015**, 119, 960–969.
3. Suchand Sangeeth, C. S.; Wan, A.; Nijhuis, C. A. Probing the nature and resistance of the molecule-electrode contact in SAM-based junctions. *Nanoscale* **2015**, 7, 12061–12067.
4. Simeone, F. C.; Yoon, H. J.; Thuo, M. M.; Barber, J. R.; Smith, B.; Whitesides, G. M. Defining the value of injection current and effective electrical contact area for EGaIn-based molecular tunneling junctions. *J. Am. Chem. Soc.* **2013**, 135, 18131–18144.
5. Salomon, A.; Cahen, D.; Lindsay, S.; Tomfohr, J.; Engelkes, V. B.; Frisbie, C. D. Comparison of electronic transport measurements on organic molecules. *Adv. Mater.* **2003**, 15, 1881–1890.
6. Akkerman, H. B. & de Boer, B. Electrical conduction through single molecules and self-assembled monolayers. *J. Phys. Condens. Matter* **2008**, 20, 013001.
7. Nitzan, A.; Galperin, M. On the electrostatic potential profile in biased molecular wires. *J. Chem. Phys.* **2002**, 117, 10837.
8. Pleutin, S.; Grabert, H. The electrostatic potential profile along a biased molecular wire: A model quantum-mechanical calculation. *J. Chem. Phys.* **2003**, 118, 3756.
9. Liang, G. C.; Ghosh, A. W.; Paulsson, M.; Datta, S. Electrostatic potential profiles of molecular conductors. *Phys. Rev. B* **2004**, 69, 115302.
10. Garrigues, A. R.; Yuan, L.; Wang, L.; Mucciolo, E. R.; Thompon, D.; de Barco, E.; Nijhuis, C. A. A Single-Level Tunnel Model to Account for Electrical Transport through Single

Molecule- and Self-Assembled Monolayer-based Junctions. *Sci. Rep.* **2016**, 6, 26517.

11. Yuan, L.; Nerngchamnong, N.; Cao, L.; Hamoudi, H.; del Barco, E.; Roemer, M.; Sriramula, R.; Thompson, D.; Nijhuis, C. A. Controlling the Direction of Rectification in a Molecular Diode. *Nat. Commun.* **2015**, 6, 6324.
12. Heimel, G.; Romaner, L.; Zojer, E.; Bredas, J. L. The Interface Energetics of Self-Assembled Monolayers on Metals. *Acc. Chem. Res.* **2008**, 41, 721–729.
13. Vilan, A.; Aswal, D.; Cahen, D. Large-Area, Ensemble Molecular Electronics: Motivation and Challenges. *Chem. Rev.* **2017**, 117, 4248–4286.
14. Malicki, M.; Guan, Z.; Ha, S. D.; Heimel, G.; Barlow, S.; Rumi, M.; Kahn, A.; Marder, S. R. Preparation and Characterization of 4′-Donor Substituted Stilbene-4-thiolate Monolayers and Their Influence on the Work Function of Gold. *Langmuir* **2009**, 25, 7967–7975.
15. Abu-Husein, T.; Schuster, S.; Egger, D. A.; Kind, M.; Santowski, T.; Wiesner, A.; Chiechi, R.; Zojer, E.; Terfort, A.; Zharnikov, M. The Effects of Embedded Dipoles in Aromatic Self-Assembled Monolayers. *Adv. Funct. Mater.* **2015**, 25, 3943–3957.
16. Cabarcos, O. M.; Shaporenko, A.; Weidner, T.; Uppili, S.; Dake, L. S.; Zharnikov, M.; Allara, D. L. Physical and Electronic Structure Effects of Embedded Dipoles in Self-Assembled Monolayers: Characterization of Mid-Chain Ester Functionalized Alkanethiols on Au{111}. *J. Phys. Chem. C* **2008**, 112, 10842–10854.
17. Cabarcos, O. M.; Schuster, S.; Hehn, I.; Zhang, P. P.; Maitani, M. M.; Sullivan, N.; Giguère, J. B.; Morin, J. F.; Weiss, P. S.; Zojer, E.; Zharnikov, M.; Allara, D. L. Effects of Embedded Dipole Layers on Electrostatic Properties of Alkanethiolate Self-Assembled Monolayers. *J. Phys. Chem. C* **2017**, 121, 15815–15830.
18. Alloway, D. M.; Graham, A. L.; Yang, X.; Mudalige, A.; Colorado, R.; Wysocki, V. H.; Pemberton, J. E.; Lee, T. R.; Wysocki, R. J.; Armstrong, N. R. Tuning the Effective

Work Function of Gold and Silver Using ω-Functionalized Alkanethiols: Varying Surface Composition through Dilution and Choice of Terminal Groups. *J. Phys. Chem. C* **2009**, 113, 20328–20334.

19. Koch, N. Organic Electronic Devices and Their Functional Interfaces. *ChemPhysChem* **2007**, 8, 1438–1455.
20. Yaffe, O.; Qi, Y.; Sheres, L.; Puniredd, S. R.; Segev, L.; Ely, T.; Haick, H.; Zuilhof, H.; Vilan, A.; Kronik, L.; Kahn, A.; Cahen, D. Charge transport across metal/molecular (alkyl)monolayer-Si junctions is dominated by the LUMO level. *Phys. Rev. B: Condens. Matter Mater. Phys.* **2012**, 85, 045433.
21. Alloway, D. M.; Hofmann, M.; Smith, D. L.; Gruhn, N. E.; Graham, A. L.; Jr., R. C.; Wysocki, V. H.; Lee, T. R.; Lee, P. A.; Armstrong, N. R. Interface Dipoles Arising from Self-Assembled Monolayers on Gold: UV−Photoemission Studies of Alkanethiols and Partially Fluorinated Alkanethiols. *J. Phys. Chem. B* **2003**, 107, 11690–11699.
22. Rusu. P. C.; Brocks, G. Work functions of self-assembled monolayers on metal surfaces by first-principles calculations. *Phys. Rev. B* **2006**, 74, 073414.
23. Ishii, H.; Sugiyama, K.; Ito, E.; Seki, K. Energy Level Alignment and Interfacial Electronic Structures at Organic/Metal and Organic/Organic Interfaces. *Adv. Mater.* **1999**, 11, 605–625.
24. Boer-Duchemin, E.; Wang, T.; Le Moal, E.; Dujardin, G. Electrically driven surface plasmon nanosources. *Proceedings of SPIE* **2015**, 9361, 93610R.
25. Schneider, N. L.; Schull, G.; Berndt, R. Optical Probe of Quantum Shot-Noise Reduction at a Single-Atom Contact. *Phys. Rev. Lett.* **2010**, 105, 026601.
26. Xu, F.; Holmqvist, C. & Belzig, W. Overbias Light Emission due to Higher-Order Quantum Noise in a Tunnel Junction. *Phys. Rev. Lett.* **2014**, 113, 066801.

27. Kaasbjerg, K.; Nitzan, A. Theory of Light Emission from Quantum Noise in Plasmonic Contacts: Above-Threshold Emission from Higher-Order Electron-Plasmon Scattering. *Phys. Rev. Lett.* **2015**, 114, 126803.
28. Persson, B. N. J.; Barato, A. Theory of photon-emission in electron-tunneling to metallic particles. *Phys. Rev. Lett.* **1992**, 68, 3224–3227.
29. Berndt, R.; Gimzewski, J. K.; Johansson, P. Inelastic tunneling excitation of tip-induced plasmon modes on noble-metal surfaces. *Phys. Rev. Lett.* **1991**, 67, 3796–3799.
30. Pechou, R.; Coratger, R.; Ajustron, F. & Beauvillain, J. Cutoff anomalies in light emitted from the tunneling junction of a scanning tunneling microscope in air. *Appl. Phys. Lett.* **1998**, 72, 671–673.
31. Kalathingal, V.; Dawson, P. & Mitra, J. Scanning tunnelling microscope light emission: Finite temperature current noise and over cut-off emission. *Sci. Rep.* **2017**, 7, 3530.
32. Downes, A.; Dumas, P. & Welland, M. E. Measurement of high electron temperatures in single atom metal point contacts by light emission. *Appl. Phys. Lett.* **2002**, 81, 1252–1254.
33. Buret, M.; Uskov, A. V.; Dellinger, J.; Cazier, N.; Mennemanteuil, M. M.; Berthelot, J.; Smetanin, I. V.; Protsenko, I. E.; Colas-des-Francs, G.; Bouhelier, A. Spontaneous Hot-Electron Light Emission from Electron-Fed Optical Antennas. *Nano Lett.* **2015**, 15, 5811–5818.
34. Zhu, Y.; Cui, L.; Natelson, D. Hot-carrier enhanced light emission: The origin of above-threshold photons from electrically driven plasmonic tunnel junctions. *J. Appl. Phys.* **2020**, 128, 233105.
35. Zhu, Y.; Cui, L.; Abbasi, M.; Natelson, D. Tuning Light Emission Crossovers in Atomic-Scale Aluminum Plasmonic Tunnel Junctions. *Nano Lett.* **2022**, 22, 8068-8075.

36. Schull, G.; Néel, N.; Johansson, P. & Berndt, R. Electron-Plasmon and Electron-Electron Interactions at a Single Atom Contact. *Phys. Rev. Lett.* **2009**, 102, 057401.

37. Fung, E. D.; Venkataraman, L. Too Cool for Blackbody Radiation: Overbias Photon Emission in Ambient STM Due to Multielectron Processes. *Nano Lett.* **2020**, 20, 8912-8918.

38. Martín-Jiménez, A.; Lauwaet, K.; Jover, O.; Granados, D.; Arnau, A.; Silkin, V. M.; Miranda, R.; Otero, R. Electronic Temperature and Two-Electron Processes in Overbias Plasmonic Emission from Tunnel Junctions. *Nano Lett.* **2021**, 21, 7086–7092.

39. Sundararaman, R., Narang, P., Jermyn, A. S., Goddard, W. A., III & Atwater, H. A. Theoretical predictions for hot-carrier generation from surface plasmon decay. *Nat. Commun.* **2014**, 5, 6788.

40. Ivashenko, O.; Bergren, A. J.; McCreery, R. L. Light Emission as a Probe of Energy Losses in Molecular Junctions. *J. Am. Chem. Soc.* **2016**, 138, 722–725.

41. Ivashenko, O.; Bergren, A. J.; McCreery, R. L. Monitoring of Energy Conservation and Losses in Molecular Junctions through Characterization of Light Emission. *Adv. Electron. Mater.* **2016**, 2, 1600351.

42. Wang, D.; Fracasso, D.; Nurbawono, A.; Annadata, H. V.; Sangeeth, C. S. S.; Yuan, L.; Nijhuis, C. A. Tuning the Tunneling Rate and Dielectric Response of SAM-Based Junctions via a Single Polarizable Atom. *Adv. Mater.* **2015**, 27, 6689.

43. Kim, K.-Y.; Kim, B. C.; Lee, H. B.; Shin, H. Nucleophilic Fluorination of Triflates by Tetrabutylammonium Bifluoride. *J. Org. Chem.* **2008**, 73, 8106.

44. Kaiser, E.; Gunther, E. P. Alcoholysis of Esters with Aluminum Alcoholates. *J. Am. Chem. Soc.* **1956**, 78, 3841.

45. Bérubé, M.; Kamal, F.; Roy, J.; Poirier, D. A Dehydrohalogenation Methodology for Synthesizing Terminal Olefins under Mild Conditions. *Synthesis* **2006**, 18, 3085.

46. Petrovykh, D. Y.; Kimura-Suda, H.; Opdahl, A.; Richter, L. J.; Tarlov, M. J.; Whitman, L. J. Alkanethiols on Platinum: Multicomponent Self-Assembled Monolayers. *Langmuir* **2006**, 22, 2578–2587.
47. Wimbush, K. S.; Fratila, R. M.; Wang, D. D.; Qi, D. C.; Cao, L.; Yuan, L.; Yakovlev, N.; Loh. K. P.; Reinhoudt, D. N.; Velders, A. H.; Nijhuis, C. A. Bias Induced Transition from an Ohmic to a Non-Ohmic Interface in Supramolecular Tunneling Junctions with Ga_2O_3/EGaIn Top Electrodes. *Nanoscale* **2014**, 19, 11246–11258.
48. Yu, X.; Wilhelmi, O.; Moser, H. O.; Vidyaraj, S. V.; Gao, X.; Wee, A. T. S.; Nyunt, T.; Qian, H.; Zheng, H. New soft X-ray facility SINS for surface and nanoscale science at SSLS. *J. Electron Spectros. Relat. Phenomena* **2005**, 144–147, 1031–1034.
49. Chen, X.; Kretz, B.; Adoah, F.; Nickle, C.; Chi, X.; Yu, X.; del Barco, E.; Thompson, D.; Egger, D. A.; Nijhuis, C. A. A single atom change turns insulating saturated wires into molecular conductors. *Nat. Commun.* **2021**, 12, 3432.
50. Yuan, L.; Breuer, R.; Jiang, L.; Schmittel, M.; Nijhuis, C. A. A Molecular Diode with a Statistically Robust Rectification Ratio of Three Orders of Magnitude. *Nano Lett.* **2015**, 15, 5506–5512.
51. Rusu, P. C.; Brocks, G. Surface Dipoles and Work Functions of Alkylthiolates and Fluorinated Alkylthiolates on Au(111). *J. Phys. Chem. B* **2006**, 110, 22628–22634.
52. Jakobi, K. 3.1.2.4 Work function data. In *Electronic and Vibrational Properties*; Chiarotti, G., Ed.; Springer-Verlag Berlin Heidelberg, **1994**.
